# Wireless bioelectronic stimulation enhanced by electroconductive silk bioink modulates human iPSC-cardiomyocyte contractility and structural organisation

**DOI:** 10.1016/j.mtbio.2026.103781

**Published:** 2026-10-24

**Authors:** Rajiv Borah, Meenakshi Suku, Dahnan Spurling, Sara Corbezzolo, Jamie Brady, Tugdual Haffner, Conor T. Buckley, Valeria Nicolosi, Michael G. Monaghan

**Affiliations:** a Discipline of Mechanical, Manufacturing and Biomedical Engineering, School of Engineering, Trinity College Dublin, Dublin 2, Ireland; b Trinity Centre for Biomedical Engineering, School of Engineering, Trinity College Dublin, Dublin 2, Ireland; c CÚRAM, Centre for Research in Medical Devices, National University of Ireland, H91 W2TY, Galway, Ireland; d Centre for Research on Adaptive Nanostructures and Nanodevices (CRANN), Trinity College Dublin, Dublin 2, Dublin, Ireland; e School of Chemistry, Trinity College Dublin, Dublin 2, Dublin, Ireland

**Keywords:** Bioelectronic interfaces, Myocardial injury, Conductive bioink, Wireless electrical stimulation, hiPSC cardiomyocyte, Silk fibroin

## Abstract

Bioelectronic interfaces can support cardiac cell function after myocardial injury by restoring disrupted electrical signalling, while enabling controlled electrical stimulation (ES). Here, we develop a pH-regulated silk fibroin/glycidoxy-silane stabilised poly(3,4-ethylenedioxythiophene):poly(styrene sulfonate) (SF/PP) bioink formulation for extrusion-printable cardiac bioelectronic interfaces. Specifically, pH neutralisation during blending moderates rapid SF-PP interactions, delays premature gelation and enables homogeneous extrusion printing. The optimised SF/PP formulations exhibit shear-thinning behaviour, continuous filament formation and stable printed grid geometries, while PP incorporation regulates hydrogel microstructure, swelling behaviour, mechanics and electrical impedance. The SF/PP bioink formulations support human induced pluripotent stem cell (hiPSC)-derived cardiomyocyte (hiCM) viability and enabled cell-laden printing of hiPSC-derived cardiac fibroblasts (hiCFs) into tubular and grid constructs with post-printing viability. When used as conductive hydrogel interfaces for capacitive-coupling wireless electrical stimulation (WES), the SF/PP formulations enhanced cardiomyocyte contraction amplitude, calcium signal propagation and sarcomeric organisation. The higher PEDOT formulation promotes more coordinated calcium propagation, while WES increases sarcomere length and is associated with higher Cx43 puncta density. These findings establish pH-regulated SF/PP bioinks as printable cardiac bioelectronic interfaces for wireless bioelectronic conditioning of human iPSC-derived cardiac cell constructs, supporting future development of electrically functional platforms for post-infarct cardiac repair studies.

## Graphical abstract

pH-regulated silk/PEDOT electroconductive bioinks are engineered to balance printability, electroactivity and soft hydrogel mechanics. The silk/PEDOT bioelectronic interface supports capacitive coupling based wireless electrical stimulation of human iPSC-derived cardiomyocytes for enhanced contractile behaviour, intercellular Ca^2+^ signalling, sarcomeric organisation and Cx43 expression.

**Figure:**
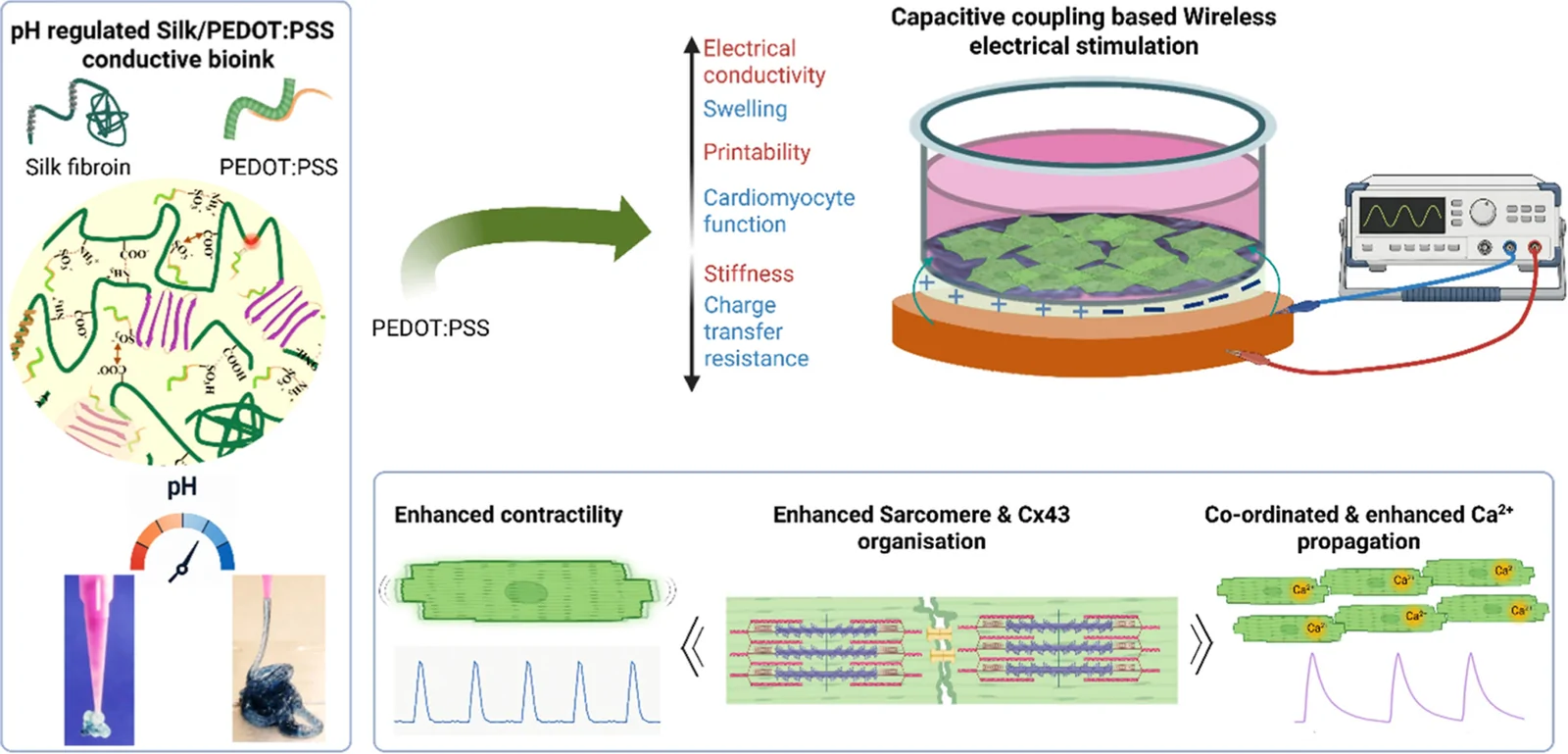

## 1 Introduction

Cardiac muscle contraction relies on the coordinated propagation of electrical signalling through an electromechanically coupled syncytium. Injury to the heart, such as myocardial infarction (MI) results in cardiomyocyte loss followed by fibrotic scar formation, which disrupts this electromechanical co-ordination. This can propagate to impedance mismatch at the scar tissue with discontinuous electrical signalling, which increases the risk of ventricular arrhythmias [1]. This has motivated the development of soft electroconductive interfaces that can restore electrical continuity across damaged tissue while minimising mechanical mismatch with the myocardium. In this context, electroconductive biomaterials that can function as soft, mechanically compliant bioelectronic interfaces, while remaining biologically permissive, have attracted sustained interest for cardiac applications since the early 2010s [2], [3]. Recent studies have demonstrated the multifunctional roles of flexible bioelectronics integrated with cardiac tissue; enabling electrophysiological recording, on-demand pacing to synchronize contraction, and controlled local drug delivery, highlighting their significant diagnostic and therapeutic potential for cardiac applications [4].

Alongside the development of conductive cardiac biomaterials, exogenous electrical stimulation (ES) has been widely investigated as an active biophysical cue to promote cardiac tissue maturation and function. ES of iPSC-derived cardiac tissues has been shown to improve myofibrillar organisation, Ca^2+^ signalling, and contractile properties [5], [6]. Conventional ES, however, typically requires direct electrode contact with the culture medium or tissue. In implantable settings, additional leads, power sources and stimulation hardware can increase device invasiveness and may necessitate secondary surgical intervention. These limitations have encouraged the development of wireless approaches for cardiac stimulation. Radiofrequency (RF)-powered passive stimulation has been shown to synchronize cardiomyocyte contraction and intracellular Ca^2+^ transients with the applied stimulation frequency in cells cultured on conductive hydrogels [7]. A subsequent RF-powered system demonstrated frequency dependent entrainment of spontaneous beating in human induced pluripotent stem cell (hiPSC) derived-cardiomyocytes (hiCMs) at 0.5-2 Hz [8]. There are also strong precedents for wireless cardiac pacing, including electromagnetic power transfer for battery-free pacing of the porcine heart [9]. Inductive coupling has similarly been used to wirelessly power fully implantable cardiac pacing systems incorporating soft conductive hydrogel electrodes [10].

Among these approaches, capacitive coupling provides a relatively simple route for wireless ES because a time-varying electric field can be transferred across an insulating dielectric without direct electrical contact between the transmitting electrode and the biological medium. This is particularly relevant for prolonged cardiac stimulation, where separation of the electrode from the culture medium can minimise unwanted electrode–electrolyte electrochemical reactions. More recently, conductive regenerative platforms have been exploited as electrical signal receiver capacitively across dielectrics. Briefly, an alternating voltage applied to an external transmitter generates a time-varying electric field across an insulating barrier (dielectric), which induces a secondary electric field in the conductive receiver. This field mobilises ionic and electronic charge carriers within the conductive biomaterial, enabling local ES of nearby cells. Wu et al. demonstrated the use of an implanted conductive chitosan/gelatin/black-phosphorus hydrogel acting as a wireless receiver, where electrostatic induction generated an alternating current within the hydrogel during high-frequency capacitive stimulation and promoted neural repair in vivo [11]. More recent work with injectable PEDOT:PSS/polypyrrole (PPy) hydrogels further demonstrated that conductivity differences between the hydrogel and surrounding tissue can promote interfacial polarisation and charge accumulation during capacitive wireless stimulation [12]. We previously established a related charge-polarisation-mediated capacitive WES approach using SF/PEDOT hydrogels, demonstrating efficient capacitive signal transduction at a substantially lower operating frequency of 10 kHz [13]. Despite these advances, the integration of capacitive WES with a printable electroconductive biomaterial interface for cardiac applications remains largely unexplored.

The use of conductive biomaterials can further enhance the efficiency of externally applied ES. For example, carbon nanotube-incorporated GelMA cardiac constructs exhibited an approximately 85% lower excitation threshold than pristine GelMA, indicating enhanced electrical coupling between the applied field and cardiomyocytes [2]. As such conductive biomaterials for cardiac tissue engineering have been developed by incorporating a range of metallic nanostructures, carbon-based nanomaterials and intrinsically conducting polymers into otherwise nature derived polymer to address the mechanical and electrical mismatch between engineered constructs and living myocardium. For example, incorporation of gold nanowires into alginate scaffolds improved electrical communication between cardiomyocytes and induced synchronous contraction [14]. Electrically conducting polymers (ECPs), owing to their high charge-transfer efficiency and mixed ionic-electronic conductivity, have been widely explored in the form of fibrous scaffolds and hydrogels for cardiac applications. For example, an injectable PPy/chitosan hydrogel was demonstrated to restore electrical signal transduction post-MI, resulting in enhanced cardiac function [15]. Recently, a mixed ionic-electronically conductive PEDOT:PSS/collagen based injectable hydrogel was shown to mitigate post-infarct arrhythmia and enhance structural and functional maturation of hiCMs [16]. These, and many other studies demonstrate that electroconductive biomaterials can restore or support electrical signalling post cardiac injury and thereby improve cardiac electromechanical function. Such injectable hydrogel formulations can conform to irregular shapes at the injury site, particularly for small defects or regions; however, they offer limited control over the spatial organisation of the resulting conductive interface. In this context, extrusion-based bioprinting offers a particularly promising additive manufacturing strategy because it enables spatial control over construct geometry, anisotropy and multi-material organisation. Recent studies show the potential of this approach for cardiac bioelectronics. For example, 3D bioprinting has been used to integrate cardiac cell-containing extracellular matrix (ECM) hydrogels with conductive and dielectric inks, producing soft cardiac patches capable of extracellular recording and electrical pacing [17]. Conductive decellularised ECM/multi-walled carbon nanotube (MWCNT) bioinks have also been printed into hiCMs laden constructs, where material conductivity and external ES improved contractile behaviour [18]. Importantly, cardiac biofabrication studies also showed cardiomyocyte seeding atop post-printing to design engineered myocardial constructs. For example, mechanically robust SF based microfibrous scaffolds have been 3D printed and subsequently seeded with neonatal cardiomyocytes and hiCMs, supporting cardiomyocyte attachment and function over extended culture periods upto 28 days [19]. Similarly, endothelial-cell-laden GelMA-alginate microfibrous scaffolds have been bioprinted and subsequently seeded with cardiomyocytes to generate synchronously contracting engineered myocardium [20]. These studies demonstrate that post-fabrication cardiomyocyte seeding represents a complementary strategy to direct cardiomyocyte encapsulation in cardiac biofabrication. However, these studies also highlight a continuing bioink-design gap. Conductive cardiac bioinks must combine print fidelity, cytocompatibility, and hydrogel stability, while maintaining a controllable gelation window that supports homogeneous mixing, continuous extrusion, and shape retention after deposition. Beyond these printing requirements, the matrix should retain wet-state electroactivity, including effective charge-transfer behaviour and capacitive performance. This is important for wireless electrical stimulation (WES), where electrical cues are delivered without placing stimulating electrodes in direct contact with the cell-containing construct. However, the integration of printability, cytocompatibility and WES-relevant electroactivity remains underexplored in printed cardiac bioelectronic systems.

Silk fibroin (SF) is a naturally derived protein with good biocompatibility, aqueous processability and tuneable physicochemical properties including biodegradability and mechanical properties [21]. SF forms hydrogels through self-assembly, involving a transition from predominantly random coil/α-helical structures to β sheet-rich physically crosslinked networks. This makes SF attractive for crosslinker-free bioink design [22]. However, SF gelation is highly sensitive to local physicochemical conditions, including protein concentration, temperature, ions and pH. Lower pH accelerates SF gelation, partly by reducing electrostatic repulsion near its isoelectric point of ∼3.9 [23]. Mechanistic studies have also shown that early-stage gelation initiates before extensive β sheet formation, highlighting the importance of weak electrostatic and hydrophobic interactions during chain association. In addition, amphiphilic molecules and polyphenolic compounds, including sodium dodecyl sulfate (SDS), sophorolipids and tannic acid, can accelerate SF gelation by modulating hydrophobic interactions, electrostatic interactions and hydrogen bonding [24], [25], [26]. For a conductive silk-based bioink, these gelation mechanisms become particularly important, for example when SF is blended with an amphiphilic PEDOT:PSS. It can therefore alter the local charge environment of SF during mixing, while also providing electronic functionality. We previously showed that PEDOT:PSS can influence SF charge balance, hydration and intermolecular association during SF/PEDOT hydrogel formation [13]. Thus, in an SF/PEDOT bioink, PEDOT:PSS is not only the electroactive component, but it can also affect gelation behaviour and microstructural homogeneity.

Here, building on our previously established crosslinker-free injectable SF/PEDOT platform [13], we translate this material concept into an extrusion-printable active bioelectronic interface. We specifically focus on three distinct but interdependent parameters for engineering this interface, namely SF concentration to enable sufficient post-deposition filament and construct stability; pH to moderate premature heterogeneous SF/PP gelation and establish a practical pre-gelation printing window; and PEDOT*GoP* content to provide electroactivity while also influencing network and extrusion behaviour. We hypothesised that regulating pH during SF/PP blending would moderate rapid SF-PP interactions sufficiently to enable homogeneous mixing and extrusion without compromising subsequent hydrogel formation. The cardiac biointerface and cell-laden processing capabilities of the resulting SF/PP platform were then evaluated through complementary in vitro experiments. Surface-seeded hiCMs were used to assess the conductive hydrogel as a receiver interface for charge-polarisation-induced capacitive WES and to examine its effects on contractility, calcium signalling and structural organisation. Separately, hiCFs were encapsulated within the optimised bioinks as a proof-of-concept assessment of cell-laden extrusion processability and short-term post-printing viability. Together, these experiments evaluate the SF/PP hydrogel as a pH-regulated printable biointerface for bioelectronic stimulation of hiCMs, while establishing a basis for future optimisation toward 3D hiCM-laden and multicellular cardiac biofabrication.

## 2 Materials and methods

### 2.1 Materials

Degummed *Bombyx mori* silk fibres were kindly gifted by the Institute of Advanced Study in Science and Technology, Guwahati, India. Lithium bromide (LiBr) and high-conductivity-grade poly(3,4-ethylenedioxythiophene):poly(styrene sulfonate) (PEDOT:PSS) were purchased from Sigma-Aldrich. The cell culture reagents for hiPSC expansion and differentiation to cardiomyocytes (iCMs) such as mTESR™ medium and CHIR99021 were purchased from Stemcell Technologies. Other cell culture reagents such as RPMI medium, B-27™ supplement, GlutaMAX, and penicillin-streptomycin (Pen-Strep) were sourced from Thermo Fisher Scientific.

### 2.2 Preparation of SF/PP bioink formulation

Silk fibroin was extracted from degummed *Bombyx mori* silk fibres [27]. Typically, degummed silk fibres were dissolved in a 9.3 M LiBr solution at 60°C for 4 h. Typically, 5 g of fibres were dissolved in 20 mL of 9.3 M LiBr solution. The resulting viscous SF solution was dialysed against distilled water for 48 h using a 12 kDa molecular-weight cut-off (MWCO) cellulose dialysis membrane, with water changes every 8 h at room temperature (RT). After dialysis, the SF solution was centrifuged at 5000 rpm for 10 min at 4°C to remove undissolved particulates, followed by filtration through a 3.1 μm syringe filter to obtain a clearer and more homogeneous solution. The final SF concentration, determined gravimetrically, typically ranged from 7 to 9 wt%. The regenerated SF solution was stored at 4°C until further use.

The high-conductivity grade commercial PEDOT:PSS dispersion was filtered through a 1.5 μm syringe filter to remove larger particulates and improve dispersion homogeneity. The filtered dispersion was then pre-stabilised with 3% (v/v) (3-glycidyloxypropyl)trimethoxysilane (GoPS) to reduce possible dissociation of the hydrophilic PSS chains from PEDOT:PSS under aqueous conditions, following our previously reported approach [28]. Briefly, GoPS-PEDOT:PSS mixture was subjected to probe sonication for 1 h followed by heating at 100°C for another 1 h. The resulting GoPS-modified PEDOT:PSS dispersion was referred to as PEDOT*GoP*.

SF/PP bioinks were prepared by blending 6 wt% SF with PEDOT*GoP* to obtain two conductive formulations containing final PEDOT:PSS concentrations of 0.12 and 0.26 wt%. These formulations were designated as SF6PP12 and SF6PP26, respectively. Pure 6 wt% SF without PEDOT:PSS was used as the non-conductive control and designated as SF6. For conductive bioink preparation, the required volume of PEDOT*GoP* was added dropwise to the SF solution, followed by the addition of a small volume of 2 M NaOH directly onto the PEDOT*GoP* droplet before mixing. PEDOT:PSS or PEDOT*GoP* is highly acidic (pH ∼3), which shifts the local pH of the SF mixture towards the isoelectric point of SF and can trigger rapid, heterogeneous gelation. Therefore, pH neutralisation was used to delay this process and enable formation of a homogeneous dark blue/black bioink. The NaOH volume was adjusted empirically, typically 1-2 μL per mL of mixture, to obtain a near-neutral formulation while avoiding visible phase separation or immediate gelation.

### 2.3 Assessment of gelation kinetics and secondary-structure evolution

Gelation kinetics of SF/PP formulations before and after the pH neutralisation were assessed using time-sweep rheology. Time-sweep rheology was performed using a rotational rheometer (Anton Paar MCR102e) equipped with a 25 mm parallel-plate geometry and a 1 mm gap. Freshly prepared SF/PP precursor mixtures (∼500 μ L with and without NaOH addition) were immediately loaded onto the lower plate, and excess material was carefully removed before the measurement. The plate temperature was maintained at room temperature (RT, ∼20-22°C) throughout the experiment. The storage modulus (G′), loss modulus (G″) and complex viscosity were recorded as a function of time under a constant strain of 0.2% and angular frequency of 10 rad/s. Measurements were performed for 60 min to monitor time-dependent viscoelastic evolution during SF/PP gelation.

Pyrene-based micropolarity analysis was used to track early local microenvironmental changes associated with SF/PP network formation, following a previously reported approach [26]. Pyrene (Sigma-Aldrich, 5 μM) was added to the 6 wt% SF solution before blending with PEDOT*GoP*. Freshly prepared non-neutralised and pH-neutralised SF/PP formulations were transferred immediately into a quartz cuvette, and fluorescence emission spectra were recorded over 70 min at an excitation wavelength of 334 nm at RT. The pyrene micropolarity ratio was calculated from the fluorescence intensity ratio between 373 and 384 nm, i.e., I_373_/I_384_. This ratio was plotted as a function of time to assess changes in the local polarity/hydrophobicity of the SF/PP microenvironment during gelation. Pyrene-free formulations were used as background controls, and all measurements were performed in triplicate.

FT-IR spectroscopy was used to evaluate SF secondary-structure evolution during gelation. Briefly, pH-neutralised SF/PP formulations were frozen at three defined time points: immediately after mixing, referred to as before gelation (BG); after 15 min at room temperature, referred to as the partially gelled stage (PG); and after incubation at 37°C for 30 min, referred to as after gelation (AG). The frozen samples were lyophilised and analysed using an ATR-FTIR spectrometer (Spectrum 100, PerkinElmer) over the 4000-750 cm^−1^ range with 1 cm^−1^ resolution. The amide I region, 1710-1595 cm^−1^, was analysed to quantify SF secondary structures. Briefly, second-derivative spectra were obtained using a third-degree polynomial function with Savitzky-Golay smoothing, followed by Gaussian peak deconvolution using OriginPro 2024. The relative contribution of each secondary-structure component was calculated by integrating the area under each deconvoluted peak and normalising it to the total amide I area. Peaks in the ranges 1695-1700, 1666-1685, 1650-1660, 1635-1645, 1610-1635 and 1600-1610 cm^−1^ were assigned to antiparallel β-sheets, loops/turns, α-helices, random coils, parallel β-sheets and side chains, respectively. β-sheet content was also quantified in lyophilised non-neutralised acidic SF/PP formulations that were frozen 15 min after preparation.

### 2.4 Rheological characterisation for extrusion printability

Rheological characterisation was performed to evaluate the extrusion-relevant flow behaviour of SF/PP hydrogel formulations. SF6PP12 and SF6PP26 formulations were analysed under two preparation conditions: non-neutralised acidic formulations and pH-neutralised formulations.

For each condition, rheological measurements were performed 15 min after formulation preparation (at RT). Steady-shear viscosity measurements were performed over a shear-rate range of 0.1-250 s^−1^. Apparent viscosity was plotted as a function of shear rate to assess shear-thinning behaviour under acidic and pH-neutralised conditions. Oscillatory amplitude-sweep measurements were performed to evaluate the stress-dependent viscoelastic behaviour and flow point of each formulation under shear strain from 0.01 to 100% at a fixed angular frequency of 10 rad/s. The storage modulus (G′), loss modulus (G″) and corresponding oscillatory shear stress were recorded throughout the test; G′ and G″ were plotted as functions of applied shear stress. The flow point was defined as the shear stress at which G′ and G″ first intersect, indicating the transition from predominantly elastic/solid-like behaviour to viscous/fluid-like flow. This crossover-based approach has previously been used as a printability-relevant rheological parameter [29]. Structural recovery after shear-induced deformation was evaluated using a three-interval time thixotropy (3ITT) cyclic step-strain recovery test. Samples were first subjected to a low strain of 1% at 1 Hz for 120 s to establish the initial viscoelastic state. A high strain of 500% at 1 Hz was then applied for 120 s to disrupt the network, followed by recovery at 1% strain and 1 Hz for 120 s. The time-dependent recovery of G′, G″ and complex viscosity were recorded to assess dynamic mechanical reversibility and post-deformation recovery of the SF/PP bioink formulations. All measurements were performed in triplicate.

### 2.5 Mechanical testing

#### 2.5.1 Static unconfined compression

Uniaxial unconfined compression testing was performed to determine the mechanical properties of SF6, SF6PP12 and SF6PP26 hydrogel constructs before and after short-term aqueous incubation. Freshly prepared pH-neutralised formulations were cut into cylindrical constructs with a diameter of approximately 10 mm and height of 3 mm. To evaluate the effect of aqueous equilibration on hydrogel mechanics, two sample conditions were tested: freshly gelled hydrogels, referred to as before incubation, and hydrogels incubated in 1×PBS (pH = 7.4) at 37°C for 24 h, referred to as after incubation. Before mechanical testing, excess surface liquid was gently removed. Compression testing was carried out using a Zwick universal testing machine (ZwickRoell, UK) equipped with a 200 N load cell. Samples were placed between parallel compression plates and subjected to a preload of 0.1 N to ensure contact between the sample and the compression platen. Hydrogels were then compressed at a constant displacement rate of 0.1 mm/s up to 80% strain at room temperature. Compressive stress was calculated by normalising the recorded force to the initial cross-sectional area of each hydrogel. The compressive modulus was determined from the slope of the linear region of the stress-strain curve. Since these hydrated hydrogel samples showed an initial non-linear toe region at low strain, the modulus was calculated from the approximately linear region between 20 and 50% strain. All measurements were performed in triplicate.

#### 2.5.2 Dynamic cyclic mechanical testing of printed SF/PP constructs

Dynamic cyclic compression was performed to evaluate the mechanical stability of printed SF/PP constructs under continuous unconfined 1-50 loading-unloading cycles between 2 and 25% compressive strain at 1 Hz, with no rest period between consecutive cycles. Printed SF6PP12 and SF6PP26 constructs measuring approximately 12 × 12 mm were fabricated as five-layer structures using the optimised printing conditions. Samples were positioned between parallel compression plates of a Zwick universal testing machine (ZwickRoell, UK) equipped with a 200 N load cell. Three independently prepared constructs were tested for each formulation (n = 3).

The data were analysed following previously established method [30], [31]. For each cycle, the force recorded at the starting strain was subtracted from the measured force to correct the small baseline offset. Compressive stress was then calculated by dividing the corrected force by the initial construct area. Peak stress was defined as the maximum corrected stress recorded during each cycle. The mechanical energy applied during loading ($U_{load}$) and recovered during unloading ($U_{unload}$) were calculated from the corresponding areas under the stress-strain curves. The dissipated energy ($U_{diss}$), corresponding to the area enclosed by the loading-unloading hysteresis loop, was calculated as:

$$U_{diss}=U_{load}-U_{unload}$$

Peak-stress retention and dissipated-energy retention for nth cycle were calculated relative to cycle 1 as:

$$\text{Peak}-\text{stress}\;\text{retention}=\frac{\sigma_{\max,n}}{\sigma_{\max,1}}\times 100$$

$$\text{Dissipated}-\text{energy}\;\text{retention}=\frac{U_{diss,n}}{U_{diss,1}}\times 100$$

Relative energy dissipation, representing the fraction of the applied loading energy dissipated within each cycle, was calculated as:

$$\text{Relative}\;\text{energy}\;\text{dissipation}=\frac{U_{diss,n}}{U_{load,n}}\times 100$$

Representative stress-strain curves from cycles 1, 5, 10, 25 and 50 were plotted, while the cycle-dependent peak-stress retention, dissipated-energy retention and relative energy dissipation were reported as mean ± SD (n = 3).

For controlled dynamic tissue deformation, chicken breast tissue was mounted in a custom-built, Arduino-controlled electromechanical tensile deformation system. SF/PP hydrogel was deposited onto the tissue surface and allowed to gel before testing. Cyclic deformation parameters were defined using a MATLAB-based control program and implemented through the Arduino-controlled actuator. The tissue-hydrogel construct was subjected to repeated tensile deformation at 25% strain and 1 Hz, while the hydrogel-tissue interface was visually monitored during loading and unloading.

### 2.6 Microstructural and porosity analysis

Scanning electron microscope (SEM, Zeiss ULTRA Plus) imaging was used to assess the porous microarchitecture of the lyophilised SF6, SF6PP12 and SF6PP26 hydrogel scaffolds. Ethanol displacement method was used for quantifying the porosity of these lyophilised scaffolds with known dimensions. The dry weight ($M_{d}$) of the scaffolds was first measured and then, they were incubated in absolute ethanol for 2 h to allow ethanol to penetrate the accessible pore network. Following incubation, the samples were removed from ethanol and excess ethanol on the external surface was gently removed without compressing the scaffold. The ethanol-saturated mass was then immediately recorded as $M_{w}$. The bulk porosity was subsequently calculated as the percentage of ethanol-accessible pore volume relative to the total geometrical volume of the scaffold:

$$Porosity\;\%=\frac{M_{w}-M_{d}}{\rho_{e}\times V}\times 100$$

$\rho_{e}$ is the density of ethanol (0.789 mg/mm^3^). Three independently prepared samples were analysed for each formulation, and the results were reported as mean ± SD.

### 2.7 Swelling study

Mass-swelling behaviour of the various hydrogel scaffolds was evaluated to assess the rehydration capacity of the lyophilised scaffold networks. Two sample conditions were first prepared: before and after incubation, as described in Section 2.5. The hydrogel formulations prepared under these conditions were lyophilised and the dry mass of each scaffold was recorded as M_d_. The dried scaffolds were then immersed in 1X PBS (pH 7.4) at 37°C for 24 h to allow rehydration. After incubation, excess surface liquid was gently removed using tissue paper, and the swollen mass was recorded as M_s_. The mass swelling ratio was calculated as M_s_/M_d_.

### 2.8 Electrical conductivity measurements

Wet-state electrical conductivity of SF6PP12 and SF6PP26 hydrogel formulations was measured using a Keithley 2400 source meter (Tektronix, USA) in a customised two-probe configuration. Conductivity was measured at two time points: at day 1 after hydrogel formation and after 20 days of incubation in 1X PBS (pH = 7.4) at 37°C. Briefly, cylindrical hydrogel samples with an approximate diameter of 6 mm and thickness of 5 mm were placed between two parallel brass electrodes. Before measurement, excess surface PBS was gently removed to minimise variability due to residual liquid at the electrode-hydrogel interface. Current-voltage (I-V) characteristics were recorded by sweeping the applied voltage from −0.5 V to +0.5 V using a step size of 40 mV/s. The surface conductivity (σ) was determined using the following formula:

$$\sigma =\frac{L}{RA}$$

Here, R and A are the resistance and cross-sectional area of the sample, whereas L is the distance between the two probes of the source meter. All measurements were performed in triplicate.

### 2.9 Electrochemical impedance spectroscopy (EIS)

For EIS measurement, SF/PP hydrogels were prepared in 1 mm thick and 3 mm diameter disks and placed onto a polished glassy carbon electrode with a 3 mm diameter, which served as the current collector for the working electrode. EIS measurements were carried out using a BioLogic VMP-300 potentiostat in a three-electrode Swagelok-type electrochemical cell in 10×PBS (pH = 7.4) from 0.01 Hz to 6 MHz, averaging 3 points per frequency, with an amplitude of 10 mV. The hydrogel-coated glassy carbon electrode was used as the working electrode, YP-50 (Kuraray) activated carbon containing 10 wt% polytetrafluoroethylene (PTFE) binder (Sigma) was used as the counter electrode, and Ag/AgCl was used as the reference electrode. A cellulose filter membrane (Whatman) was placed between the working and counter electrode compartments as a separator.

### 2.10 Printability characterisation

Extrusion printability of pH-neutralised SF6PP12 and SF6PP26 formulations was evaluated using a CELLINK BIO X™ pneumatic extrusion bioprinter. Prior to printing, the SF and PEDOT*GoP* precursor solutions were filtered as described in Section 2.2 to remove particulates and minimise nozzle blockage. Freshly prepared pH-neutralised SF/PP bioinks were loaded into 3 mL printing cartridges and extruded through a 22 G tapered dispensing tip (Fisnar). Printing was performed under pneumatic pressure control, and the printing pressure was varied across two predefined pressure windows, 45-55 kPa and 56-70 kPa, to identify formulation-specific extrusion and shape-fidelity behaviour.

Printability was evaluated using quantitative shape-fidelity metrics adapted from previously reported extrusion-bioprinting approaches, including uniformity factor (*UF*), spreading ratio (*SR*), angle fidelity factor (*AF*), and printability index (*Pr*) [32]. Straight-line filaments were first printed to evaluate filament continuity, uniformity and lateral spreading. Images of the printed filaments were acquired immediately after printing and analysed using Fiji. The uniformity factor was determined using the following formula:

$$UF=\frac{L_{\exp}}{L_{th}}$$

where *L*_exp_ is the experimentally measured length of the extruded filament and *L*_*th*_​ is the theoretical designed filament length.

The spreading ratio was calculated from the same printed filaments as:

$$SR=\frac{t_{filament}}{d_{needle}}$$

where *t*_*filament*_ is the measured filament width after deposition and *d*_*needle*_ is the inner diameter of the printing tip.

*AF* was evaluated by printing angular geometries at 90° and 45° to assess pressure-dependent corner accuracy and material accumulation at the vertex. The angle fidelity factor was determined as:

$$AF=\frac{t_{tip}}{t_{f}}$$

where *t*_*tip*_ is the filament thickness at the angle vertex and *t*_*f*_ is the average filament thickness along the straight segment.

To assess pore-level shape fidelity, square grid constructs were printed and analysed from optical images. The Pr was calculated from the geometry of printed pores using:

$$\mathrm{Pr}=\frac{L^{2}}{16A}$$

where *L* is the measured pore perimeter and *A* is the enclosed pore area.

A filament collapse test was performed following a previously reported protocol [33]. Briefly, single filaments were deposited across a custom-designed platform containing parallel supports separated by defined gap distances of 1, 2, 4, 8 and 16 mm. SF6PP26 was printed in a pressure window of 45-50 kPa, while SF6PP12 was printed at a slightly higher pressure of 52-55 kPa. Printed filaments were imaged immediately after deposition, and the deflection angle (θ) at each gap was measured using Fiji. Lower θ values indicated better resistance to filament sagging and improved shape fidelity. Data were reported as mean ± SD from multiple printed filaments.

### 2.11 Human iPSC derived cardiomyocyte differentiation

Human iPSC-derived cardiomyocytes (hiCMs) were generated using small-molecule modulation of Wnt/β-catenin signalling based on the GiWi differentiation protocol [34]. Briefly, SFCi55 hiPSCs were expanded in mTeSR medium (Stemcell technologies) on Matrigel-coated culture plates until approximately 90-100% confluent. On day 0, cells were pre-treated with 10 μM ROCK inhibitor (Y-27632, Stemcell technologies) for 1 h, after which mesoderm induction was initiated by replacing the maintenance medium with RPMI 1640 medium (Gibco) supplemented with GlutaMAX, 2% penicillin-streptomycin and 2% B27 supplement without insulin (RPMI-i) containing 10 μM CHIR99021 (Stemcell Technologies). After 24 h, CHIR99021-containing medium was removed, cells were washed with RPMI medium to remove dead cells, and fresh RPMI-i medium was added. On day 3, RPMI-i was supplemented with 5 μM IWP-2 (Sigma) to inhibit Wnt signalling and promote cardiac mesoderm specification. Following 48 h of IWP-2 treatment, on day 5, the medium was replaced with fresh RPMI-i. From day 7 onwards, cells were maintained in RPMI 1640 medium supplemented with GlutaMAX, 2% penicillin–streptomycin and 2% B27 supplement containing insulin (RPMI + i), with medium changes every 3 days. Spontaneous beating was typically observed from days 8-12. Following differentiation, hiCMs were subjected to lactate-based metabolic purification using glucose-free RPMI 1640 medium supplemented with 4 mM lactate for 5 days. After purification, cells were returned to RPMI + i medium for further maturation. Purified hiPSC-CMs were used for downstream experiments after further maturation in culture for another one or two weeks.

### 2.12 Human iPSC derived cardiac fibroblast differentiation

Human iPSC-derived cardiac fibroblasts (hiCFs) were generated from SFCi55 iPSCs using a previously established Wnt/FGF-based differentiation protocol [35]. Briefly, differentiation was initiated at day 0 by mesoderm induction using 10 μM CHIR99021. Before 72 h of mesoderm induction, the medium was replaced with complete FibroGRO™ medium (Sigma) supplemented with 75 ng/mL fibroblast growth factor 2 (FGF2). Cells were maintained, with medium changes every other day, and cultured for 20 days. At day 20 of differentiation, cells were considered hiCF progenitors and were passaged into tissue-culture-treated T75 flasks or 6-well plates; passaged cells were subsequently referred to as hiCFs.

### 2.13 hiPSC cardiac fibroblast laden 3D bioprinting

For cell-laden 3D bioprinting, sterile SF6PP12 and SF6PP26 formulations were freshly prepared and pH-adjusted using 1-2 μL of 2 M NaOH per mL of formulation. Before cell incorporation, 100 μL of high-glucose DMEM containing 10% FBS was gently mixed with 500 μL of the freshly prepared SF/PP formulation. This increased the pH of the resulting bioink mixture to approximately 6.7-7.2. A predetermined hiCF pellet (passage 4-6) was then gently resuspended directly into the medium-supplemented SF/PP formulation to obtain a final cell density of 2 × 10^6^ cells/mL. The cell-laden bioinks were transferred into sterile 3 mL printing cartridges and printed at room temperature (20-22°C) using a CELLINK BIO X™ pneumatic extrusion bioprinter fitted with a 22 G tapered dispensing tip. Printing was performed within 15 min of hydrogel preparation using formulation-specific pneumatic pressures of 64-70 kPa for SF6PP12 and 45-55 kPa for SF6PP26. Cell-laden SF6PP12 and SF6PP26 bioinks were printed into circular/tubular (∼8 mm diameter) and grid-shaped constructs (approximately 12 mm × 12 mm). After printing, constructs were incubated at 37°C to allow hydrogel stabilisation and were then maintained in hiCF culture medium under standard cell-culture conditions.

### 2.14 Live/dead staining and quantitative analysis

Live/dead staining was performed to assess cell viability in both hiCF-laden printed SF/PP bioink constructs and hiCMs cultured on SF/PP hydrogel surfaces. For bioprinted hiCF constructs, samples were stained at 12, 24 and 96 h after printing using a staining solution containing 2 μM Calcein-AM (Biotium) and 4 μM ethidium homodimer-1 (EthD-1, Abcam) prepared in 1x PBS. Samples were incubated with the staining solution for 1 h at 37°C and 5% CO_2_, protected from light. After staining, constructs were gently washed with PBS and imaged using an Olympus fluorescence microscope. Viable cells were identified by green Calcein-AM fluorescence, while dead cells were identified by red EthD-1 fluorescence. For hiCM viability assessment, cells were seeded onto SF6, SF6PP12 and SF6PP26 hydrogel surfaces at a density of approximately 2 × 10^5^ cells/cm^2^. Live/dead staining was performed after 7 days of culture using the same Calcein-AM/EthD-1 staining procedure as described above.

For quantitative analysis of hiCF viability in the bioprinted constructs, fluorescence images were analysed using Fiji software. The green Calcein-AM live-cell channel and red EthD-1 dead-cell channel were separated and processed independently. Images were converted into binary masks using a consistent thresholding strategy across images from the same experiment. For the Calcein-AM-positive live-cell channel, particles with an area of 100-5500 μm^2^ were included, while for the EthD-1-positive dead-cell channel, particles with an area of 80-800 μm^2^ were included to quantify dead-cell-associated signal. The total Calcein-AM + ve and EthD-1 +ve areas were quantified using the “Analyse Particles” function. Live-cell (or dead cell) coverage was calculated as the percentage of Calcein-AM + ve area (or EthD-1 +ve area) normalised to the total printed construct area. At least five representative images were analysed per group, and data were presented as mean ± SD.

### 2.15 MTT assay

Metabolic activity of hiCMs cultured on SF/PP hydrogel surfaces was evaluated using an MTT assay. Briefly, hiCMs were seeded onto freshly prepared or media-preconditioned SF6, SF6PP12 and SF6PP26 hydrogel surfaces (cell density ∼ 2 × 10^5^ cells/cm^2^) and cultured for 3 and 7 days. At each time point, samples were gently washed twice with sterile 1X PBS and incubated with culture medium containing 0.5 mg/mL of MTT reagent for 3 h at 37°C and 5% CO_2_, protected from light. The MTT stock solution was prepared by dissolving thiazolyl blue tetrazolium bromide in sterile 1X PBS at 5 mg/mL, which was then diluted in culture medium to obtain the final concentration of 0.5 mg/mL. After incubation, the MTT solution was removed and samples were washed twice with 1x PBS, followed by the addition of dimethyl sulfoxide (DMSO) to dissolve the formazan crystals (produced by metabolically active cells) under constant shaking for 15 min. The solubilised formazan solution was transferred into a clear flat-bottom 96-well plate in triplicate, and absorbance was measured at 570 nm using a microplate reader. Acellular hydrogel samples treated with DMSO were used as background controls, and the corrected absorbance values were reported as mean ± SD.

### 2.16 Capacitive coupling based wireless electrical stimulation of hiCMs via SF/PP bioink hydrogels

Prior to cell seeding, SF6PP12 and SF6PP26 hydrogel constructs were prepared in glass-bottom culture dishes and preconditioned in RPMI-based culture medium for 24 h at 37°C. hiCMs were then seeded (cell density ∼ 2 × 10^5^ cells/cm^2^) onto the hydrogel surfaces and maintained under standard culture conditions before stimulation. For capacitive-coupling-based wireless electrical stimulation (WES) of hiCMs, a copper transmitter electrode was positioned beneath the culture dish, while the glass-bottom dish acted as a dielectric barrier between the transmitter and the hydrogel-containing culture medium. The conductive SF/PP hydrogel served as the receiver interface. A time-varying electrical signal was applied to the copper transmitter using a function generator, allowing capacitive coupling through the dielectric barrier without direct electrode contact with the culture medium or cells. This configuration was used to induce charge polarisation and displacement-current-mediated ES within the conductive hydrogel receiver. A burst-modulated biphasic square-wave signal was used for cardiomyocyte stimulation. Specifically, a 10 kHz carrier signal with an amplitude of 18 Vpp (±9 V) was delivered in 5 ms bursts at a burst repetition frequency of 2 Hz. Under this configuration, each 5 ms burst contained approximately 50 carrier cycles. The electric field at the hydrogel interface was approximately 0.8-1.2 V/cm as measured by a digital oscilloscope. Non-stimulated control groups were cultured on the same hydrogel formulations and handled in parallel without application of the WES. Signal transmission through the capacitive-coupling setup was first verified using a benchtop LED demonstration, in which burst packets transmitted through the dielectric barrier powered an LED connected to the conductive receiver. For biological experiments, hiCMs cultured on SF6PP12 and SF6PP26 hydrogels were subjected to WES according to the defined stimulation regime, and downstream contractility, calcium signalling and immunocytochemical analyses were performed as described in the subsequent sections. Microenvironmental temperature monitoring during WES and electrothermal finite-element modelling (FEM) of the experimental configuration were performed as described in the Supporting Information [Sections S3 & S4].

### 2.17 Intracellular Ca^2+^ signalling assessment

Intracellular Ca^2+^ signalling in hiCMs cultured on SF6PP12 and SF6PP26 hydrogels, with or without WES, was assessed on day 8 using Cal-520 AM dye (Abcam, ab171868). WES was applied according to the defined stimulation regime as mentioned above from day 3 to day 8 continuously. Cal-520 a.m. stock solution was prepared at 5 mM and diluted directly into RPMI + i medium to obtain a final working concentration of approximately 3 μM. Samples were incubated with Cal-520 a.m.-containing medium for 90 min at 37°C and 5% CO_2_, protected from light. After incubation, the staining medium was removed, samples were gently washed with PBS, and fresh RPMI + i medium was added before live-cell imaging.

Live Ca^2+^ imaging was performed using an Olympus IX81 inverted epifluorescence microscope. Fluorescence videos were acquired from spontaneously beating hiCM regions on SF6PP12 and SF6PP26 hydrogels under non-stimulated and WES-treated conditions. Multiple regions of interest (ROIs) were selected from each video to extract time-dependent fluorescence intensity traces. For quantitative Ca^2+^ propagation analysis, two spatially separated ROIs were selected along the apparent propagation direction in each recording. For each ROI, raw fluorescence intensity was normalised using the following equation:

$$Normalised\;fluorescence\;intensity=\frac{F-F_{\min}}{F_{\max}-F_{\min}}$$

where *F*_min_ and *F*_max_ represent the minimum and maximum fluorescence intensity values for that ROI during the recording. Normalised traces were used to evaluate Ca^2+^ transient regularity and inter-regional synchrony. Ca^2+^ propagation delay was calculated as the time difference between corresponding Ca^2+^ transient peaks in two spatially separated ROIs. Propagation velocity was calculated by dividing the measured inter-ROI distance by the corresponding time delay. Three independent hiCM-seeded hydrogel samples were analysed per experimental group (n = 3), with at least seven corresponding Ca^2+^ transients evaluated per ROI pair in each recording. The individual time delays and propagation velocities were averaged within each recording to obtain one value per independent sample. Data are presented as mean ± SD across the three independent samples.

### 2.18 MUSCLEMOTION video analysis

Contractile activity of hiCMs cultured on SF6PP12 and SF6PP26 hydrogel surfaces, with or without WES, was assessed on day 8 and also day 28 by bright-field video analysis using the MUSCLEMOTION plugin for Fiji [36]. Videos of spontaneously beating hiCM regions were acquired using an Olympus inverted microscope at a frame rate of 15 frames/second. Videos were imported into Fiji and analysed using the MUSCLEMOTION plugin to quantify contraction-associated pixel displacement over time. For analysis, automatic reference-frame detection and automatic transient detection were applied. The speed window was set to 2, and contraction peaks were detected from the generated motion traces using the plugin's automatic peak-detection workflow. From each video, MUSCLEMOTION-derived traces were used to calculate beat rate, relative contraction amplitude and mean peak contraction speed. Beat rate was calculated from the number of detected contraction peaks per minute, relative contraction amplitude was used as an index of contraction-associated motion magnitude, and mean peak contraction speed was used to assess contraction kinetics. Data were reported as mean ± SD from multiple ROIs per group.

### 2.19 Immunocytochemistry

Immunocytochemistry was performed to assess hiCM phenotype, sarcomeric organisation and Cx43-associated gap-junction expression on SF/PP hydrogel formulations with or without WES. The culture duration was 28 days and WES was applied from day 21 to day 28. Briefly, hiCM-seeded SF6PP12 and SF6PP26 hydrogel samples (cell density ∼ 2 × 10^5^ cells/cm^2^) were gently washed with 1X PBS and fixed with ice-cooled methanol for 5 min. Samples were then washed three times with 1X PBS, permeabilised and blocked using 4% BSA in 1X PBS containing 0.1% Triton X-100 and 0.05% Tween-20 for 45 min at room temperature. For cardiomyocyte phenotype and sarcomeric organisation, samples were incubated overnight at 4°C in a humidified chamber with mouse anti-sarcomeric α-actinin antibody (Invitrogen, MA1-22863; 1:200) and Alexa Fluor® 647-conjugated rabbit anti-cardiac troponin T antibody (Abcam, ab314677; 1:200). Following washing, samples were incubated with goat anti-mouse IgG Alexa Fluor 488 secondary antibody (Abcam, ab150113; 1:1000) for 2 h at room temperature in the dark to detect α-actinin. For sarcomeric organisation and gap-junction staining, samples were incubated overnight at 4°C with mouse anti-sarcomeric α-actinin antibody (Invitrogen, MA1-22863; 1:200) and rabbit anti-connexin-43 antibody (Sigma-Aldrich, C6219; 1:1000) prepared in working buffer. The following day, samples were washed three times with 1X PBS containing 0.05% Tween-20 and incubated for 2 h at room temperature in the dark with goat anti-mouse IgG Alexa Fluor 488 secondary antibody (Abcam, ab150113; 1:1000) and donkey anti-rabbit IgG Alexa Fluor 594 secondary antibody (Abcam, ab150076; 1:1000). Nuclei were counterstained with DAPI in 1X PBS. Fluorescence images were acquired using a Leica SP8 scanning confocal microscope.

### 2.20 Image analysis for sarcromeric length and Cx43 expression

Quantitative image analysis of sarcomeric organisation and Cx43 expression was performed using Fiji on confocal images of day 28 hiCMs cultured on SF6PP12 and SF6PP26 hydrogels, with or without WES. Sarcomere length was quantified from high-magnification α-actinin-stained images [37]. Clearly resolved, striated α-actinin-positive myofibrils were selected, and sarcomere length was measured manually as the distance between adjacent α-actinin-positive Z-bands along the longitudinal axis of individual myofibrils. Multiple measurements were taken from each image and at least 6 images were analysed.

Cx43 expression was quantified using an ROI based workflow. For each image, the α-actinin-positive cellular region was manually defined as the ROI based on the visible cell boundary, excluding signal outside the selected region. Images were spatially calibrated using the 20 μm scale bar to convert pixel area into μm^2^. Cx43-positive structures were segmented from the red channel after background reduction and thresholding, with identical thresholding settings applied across comparable images to generate binary masks of Cx43-positive signal. Total Cx43 +ve area within each ROI was measured and normalised to the number of DAPI-positive cells within the same ROI, and reported as Cx43-positive area per cell (μm^2^/cell). For puncta analysis, connected Cx43+ve objects within each ROI were identified using the “Analyse Particles” function. Objects with an area >1 μm^2^ and ≤10 μm^2^ were classified as discrete Cx43 puncta, while smaller speckles and larger diffuse or aggregated regions were excluded. Cx43 puncta density was calculated by normalising the number of accepted Cx43- + ve puncta to the analysed hiCM ROI area using the following equation:

$$Cx43\;puncta\;density=\frac{Number\;of\;Cx43+ve\;puncta}{hiCM\;sarcomeric\;actinin+ve\;area}\times 1000$$

The results are reported as puncta per 1000 μm^2^ hiCM area. Annotated overlays were generated to verify that accepted puncta were located within the selected cellular ROI. Quantification was performed on raw single-channel confocal images using consistent thresholding settings, and data from at least five images were used for analysis.

### 2.21 Statistical analysis

Statistical analysis was performed using GraphPad Prism 10 software. Quantitative data are presented as mean ± SD, unless otherwise stated. All experiments were performed with a minimum of three independent replicates, and the exact number of replicates for each assay is indicated in the corresponding figure legends. For comparisons between two groups, an unpaired two-tailed Student's t-test was used where appropriate. For comparisons among two or more groups, one-way analysis of variance (ANOVA) followed by Tukey's multiple-comparisons post hoc test was applied. For datasets involving two independent variables, such as formulation and pressure window or formulation and stimulation condition, two-way ANOVA followed by appropriate multiple-comparisons testing was used. Statistical significance was defined as p < 0.05. Significance levels are indicated as p < 0.05 or 0.01 or 0.001 or p < 0.0001; non-significant comparisons are not shown.

## 3 Results and discussion

### 3.1 pH driven electrostatic interactions delay sol-gel transition in SF/PP biomaterial ink for extrusion printing

SF undergoes a sol-gel transition through self-assembly and a progressive conformational conversion from random coil/α-helical structures to β-sheet rich physically crosslinked networks. This process is highly sensitive to pH because it regulates SF charge state, hydration and intermolecular association. At lower pH, reduced electrostatic repulsion promotes partial dehydration and association of hydrophobic domains, accelerating β-sheet formation and shortening gelation time. In contrast, near-neutral pH conditions can maintain greater electrostatic stabilisation and slow network formation [22], [23]. Beyond pH, SF gelation can also be accelerated by amphiphilic or polyphenolic molecules, including SDS, sophorolipids and tannic acid, which modulate hydrophobic interactions, electrostatic association and hydrogen bonding during network formation [24], [26], [38]. These molecules can interact with the hydrophobic SF domains, altering local charge presentation and creating multivalent non-covalent interaction sites, thereby promoting chain association, β-sheet nucleation and network consolidation during gelation.

These mechanisms are directly relevant to SF/PP bioink formulation as PEDOT*GoP* retains an amphiphilic, polyanionic character, with hydrophobic PEDOT rich domains and sulfonated PSS-rich domains (-SO_3_^-^). In our previously reported injectable SF/PP hydrogel system, SF and PEDOT:PSS interactions produced a partially gelled formulation suitable for injection after incubation at physiological temperature, followed by maturation into a mechanically robust gel [13]. For extrusion printing, however, the same interactions must be temporally controlled to preserve a homogeneous and extrudable pre-gel state while retaining sufficient post-deposition gelation for shape fidelity. The acidic PEDOT*GoP* dispersion (pH∼3) lowers the local pH during blending, and thereby drives rapid SF/PP complexation, heterogeneous gelation and phase separation unless the pH is controlled [Fig. 1(B) (i & ii)]. We therefore used PEDOT*GoP* as a dual-function component that provides electroactivity, while also influencing SF gelation, and applied pH neutralisation during blending to moderate early SF/PP interactions and create a reproducible printing window [Fig. 1(A)].

**Fig. 1:**
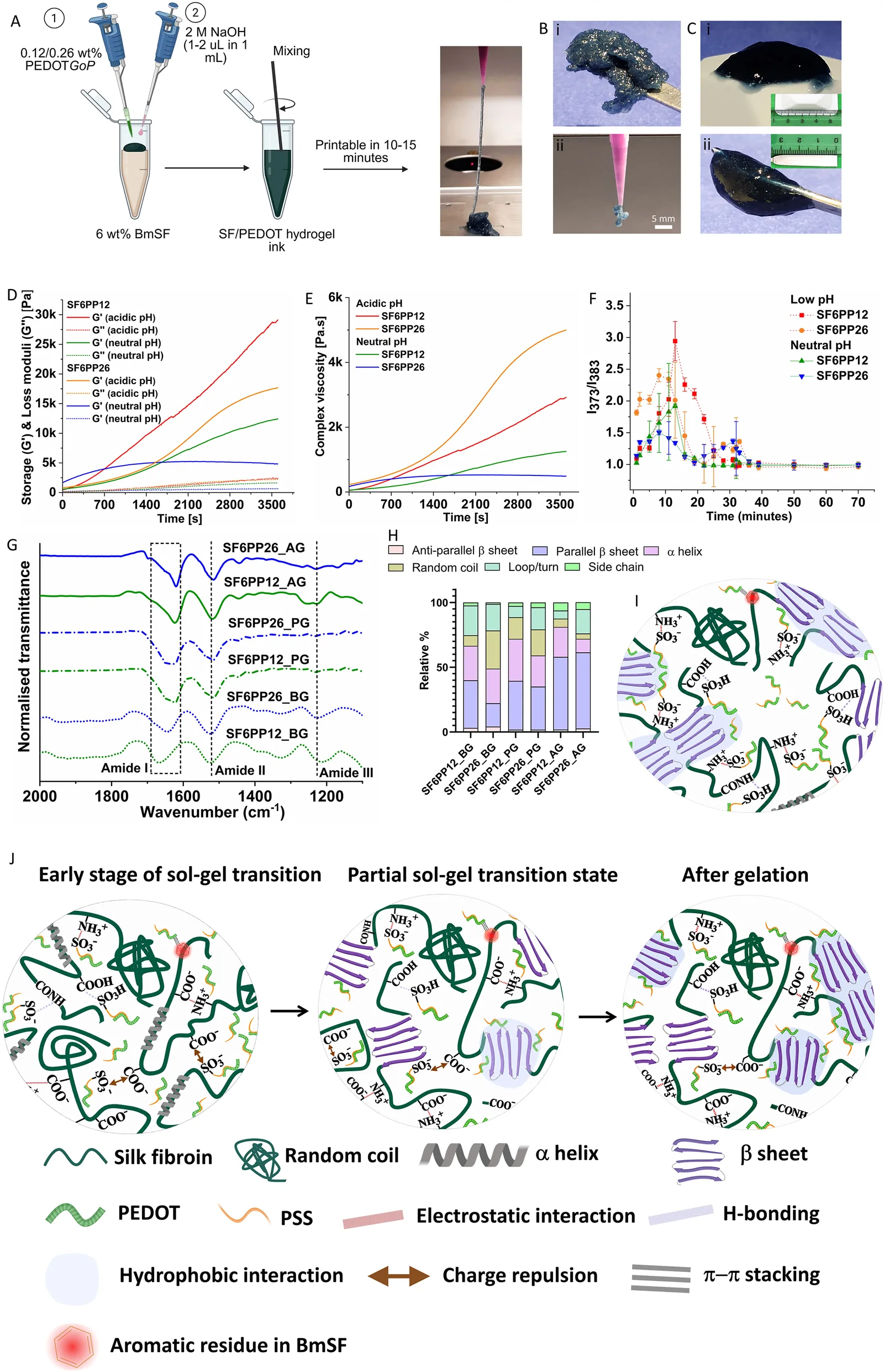
**pH-tuneable electrostatic interactions regulate SF/PP hydrogel gelation and establish an extrusion-printing window.** (A) Schematic of bioink formulation using 6 wt% *Bombyx mori* silk fibroin (BmSF) and GoPS-modified PEDOT:PSS (PEDOTGoP, 0.12 or 0.26 wt%), followed by addition of 2 M NaOH (1–2 μL mL^−1^). Acidic PEDOTGoP induces rapid, heterogeneous gelation [B(i–ii)], whereas pH neutralisation yields a homogeneous SF/PP ink [C(i–ii)] printable for ∼10–15 min. Scale bar in B(ii) = 5 mm. For dimensional reference, the weighing boat in C(i) is ∼43 × 43 mm and the stainless-steel spatula in B(i)/C(ii) is ∼15 cm long; ruler-reference images are shown as insets. (D-E) Time-sweep rheology showing faster modulus and viscosity development under acidic conditions than after pH neutralisation. (F) Pyrene fluorescence index (I_373_/I_383_) during gelation; mean ± SD, n = 3. (G) FT-IR spectra before (BG), during (PG), and after gelation (AG). (H) Amide I deconvolution showing progressive β-sheet enrichment. (I) Proposed mechanism showing how acidic conditions promote strong SF-PEDOT:PSS association (reduced electrostatic repulsion and enhanced non-covalent interactions), accelerating chain rearrangement and β-sheet “locking” into a percolated gel; (J) pH neutralisation moderates these interactions, delaying β-sheet formation to provide a transient, printable sol state prior to full gel maturation, consistent with non-covalent interaction-driven SF gelation behaviour.

We used 6 wt% of regenerated SF solution to blend with PEDOT*GoP* at 0.12 or 0.26 wt%, to prepare the bioink formulations, namely SF6PP12 and SF6PP26, respectively. When acidic PEDOT*GoP* is mixed with SF without pH control, the blend undergoes instantaneous, heterogeneous gelation/phase separation, yielding an inhomogeneous composite, which was not extrudable [Fig. 1(B)(i & ii), Fig. S1(A)]. In contrast, NaOH-mediated pH adjustment produced homogeneous SF/PP formulations that remained readily extrudable within a practical ∼10-15 min printing window [Fig. 1(C)(i & ii), Fig. S1(B)].

To further clarify the effect of pH adjustment on the processing window, early SF/PP network formation was monitored by turbidity changes at 550 nm using 1 or 2 μL of 2 M NaOH per mL of formulation following previous studies [39]. Without NaOH addition, the SF/PP formulations remained acidic (∼pH 4-5) and underwent rapid heterogeneous gelation, preventing reproducible pipetting for absorbance measurement. Addition of 1 and 2 μL/mL NaOH increased the pH to ∼5.93-6.03 and ∼6.67-6.92, respectively, and enabled time-resolved measurements. Both NaOH-adjusted formulations showed a progressive increase in the absorbance values at 550 nm over 30 min [Fig. S2], consistent with increasing light scattering during SF/PP aggregation and network formation. Increasing the NaOH dose from 1 to 2 μL/mL moderated this turbidity increase, particularly for SF6PP26, indicating slower network development and an extended pre-gel processing state. Consistent with this trend, increasing the NaOH dose to 3 μL/mL increased the formulation pH to ∼7.5, while further increases beyond this level produced a pronounced qualitative delay in gelation, with some formulations remaining in a pre-gel state for approximately 12-24 h as observed during preliminary formulation optimisation stage. This dose-dependent behaviour is consistent with the established pH sensitivity of SF gelation [23]. Pure SF6 did not undergo complete macroscopic gelation over the same 30 min period and showed slower turbidity development. Collectively, these observations demonstrate a clear NaOH-dose-dependent modulation of SF/PP gelation, with increasing formulation pH progressively extending the pre-gel processing state.

Rapid, inhomogeneous, phase separated solidification of the SF/PP composite under acidic conditions is supported by the rapid increase in storage modulus and complex viscosity during oscillatory time sweep rheology [Fig. 1(D & E)]. In contrast, pH neutralisation markedly slowed the modulus and viscosity build-up, yielding a relatively longer gelation window. Notably, across both conditions the storage modulus (G′) remained higher than the loss modulus (G″), indicating predominantly elastic, solid-like evolution during gelation, but the rate of transition was strongly suppressed under pH neutralised conditions. Pyrene micropolarity analysis further supported this pH-dependent difference in early network formation. Pyrene micropolarity index (I_373_/I_383_) displayed a sharp but transient increase in polarity at low pH, consistent with rapid microstructural rearrangement and pyrene redistribution [Fig. 1(F)]. Control measurements showed minimal time-dependent changes in dH_2_O, PEDOT:PSS and PEDOT*GoP* alone, whereas SF showed a transient pyrene response, indicating that the SF/PP micropolarity changes mainly reflect SF-associated microenvironmental rearrangement rather than pyrene drift or PEDOT*GoP* alone [Fig. S3, Supporting Information]. During rapid and aggressive electrostatic interactions between SF and PEDOT*GoP*, microstructural rearrangement in SF network (self-assembly of hydrophobic domains), pyrene may be displaced from hydrophobic pockets into a more aqueous environment, which raises the I_373_/I_383_ ratio. Then, as soon as the system consolidates (aggregation/network formation), pyrene can be trapped into the hydrophobic domains, the ratio drops back. Such rapid micropolarity changes are in agreement with SDS induced SF gelation [26]. In contrast, this early micropolarity spike was largely absent under pH-neutralised conditions, indicating slower SF/PP microstructural rearrangement and a more controlled interaction regime that preserved the printable sol-gel window.

To confirm whether the pH-tuned delay in viscoelastic build-up is accompanied by an expected silk secondary structure conformational evolution during network formation, we monitored the secondary structure of SF using FT-IR at three defined stages: before gelation (BG), partial gelation (PG; within the printing window), and after gelation (AG) for both SF6PP12 and SF6PP26 prepared at neutral pH condition [Fig. 1 (G & H)]. Across all conditions, the spectra were dominated by characteristic SF amide I, II, and III bands [Fig. 1(G)], consistent with SF being the major constituent of the composite; characteristic PEDOT:PSS vibrational features largely overlap with these SF peaks in the amide region, as observed previously for SF/PP hydrogels [13]. As gelation progressed from BG to PG and AG, the amide I region (≈1705-1590 cm^−1^) showed progressive redistribution toward the lower-wavenumber β-sheet-associated region, particularly around ∼1610-1635 cm^−1^, consistent with increasing β-sheet organisation during SF gel maturation. Amide I peak deconvolution further showed an overall increase in the relative contribution of β-sheet-associated structures during gel maturation [Fig. 1(H) and Fig. S5]. The temporal evolution was formulation dependent: SF6PP26 showed increased β-sheet contribution from BG through PG to AG, whereas SF6PP12 showed only a slight increase from BG to PG followed by a marked increase at AG. In both formulations, β-sheet-associated structures became dominant after gelation, supporting consolidation of the SF network through β-sheet-mediated physical crosslinking.

In addition to evaluating SF secondary-structure evolution, the GOPS modification of PEDOT:PSS was independently examined by comparative FT-IR and EDX analyses [Fig. S4, Supporting Information]. Compared with unmodified PEDOT:PSS, PEDOT*GoP* showed a more pronounced feature around ∼1100 cm^−1^, attributable to Si-O-Si stretching, together with spectral changes around ∼950 cm^−1^ associated with silane/siloxane vibrations [28]. Complementary EDX analysis revealed a distinct Si signal in PEDOT*GoP* that was absent from unmodified PEDOT:PSS, while Si was also detectable after incorporation of PEDOT*GoP* into the SF6PP12 composite. GOPS crosslinking of PEDOT:PSS was reported to occur predominantly through reaction of the GOPS epoxy group with sulfonic acid/sulfonate groups of PSS, while the methoxysilane groups undergo condensation to form Si-O-Si linkages [40]. The Si-O-Si-associated FT-IR feature observed here is therefore consistent with this established crosslinking mechanism.

Based on the time-sweep rheology, pyrene micropolarity and FT-IR results, we propose a pH-regulated SF/PP gelation mechanism both under acidic [Fig. 1(I)] and pH neutralised conditions [Fig. 1(J)]. Under acidic conditions, PEDOT*GoP* accelerates SF/PP gelation by reducing SF charge stabilisation and promoting rapid non-covalent association. Protonation of SF carboxylate groups at low pH (-COO^-^ to -COOH) reduces electrostatic repulsion and favours hydrophobic-domain association, while anionic PSS-rich domains (-SO_3_^-^) can interact with positively charged SF regions, including protonated amine groups (-NH_3_^+^). Together with hydrogen bonding, hydrophobic interactions and possible π–π interactions between aromatic SF residues and PEDOT-rich domains, these effects drive rapid SF/PP complexation, premature network consolidation and β-sheet locking, producing heterogeneous phase-separated gels [Fig. 1(I)]. In contrast, pH neutralisation maintains greater SF hydration and charge repulsion through deprotonated carboxylate groups (-COOH to -COO^-^). Such strengthened electrostatic repulsion between SF chains and repulsion between negatively charged SF regions and PSS-rich domains may additionally reduce SF-PEDOT*GoP* interaction. This progressively slows network percolation while still allowing subsequent β-sheet-mediated gel maturation after deposition [Fig. 1(J)], thereby providing a tunable pre-gel window for extrusion printing.

Following the pH-dependent gelation behaviour as shown in Fig. 1 (i.e., pH neutralisation delays premature network percolation and preserves a practical printing window), we next evaluated whether the resulting SF/PP formulations exhibit the flow profile required for extrusion printing. Both SF6PP12 and SF6PP26 displayed shear-thinning behaviour, with viscosity decreasing as shear rate increased under both acidic and neutralised conditions [Fig. 2 (A & B)]. This feature is essential for printability because it enables the ink to flow readily through the nozzle under high shear, while maintaining a much higher apparent viscosity at low shear after deposition to support filament formation and shape retention. Importantly, pH neutralisation showed a significant shift toward lower shear viscosity (from order of 10^6^ to order of 10^4^) across the printing-relevant shear-rate range, indicating improved extrudability compared with the corresponding acidic formulations. This pH neutralisation mediated viscosity reduction is consistent with our previous observation in Fig. 1 (E). Amplitude-sweep measurements further showed that under acidic conditions both SF6PP12 and SF6PP26 had significant increase in storage modulus (Gʹ∼7000-10000 Pa) in contrast to their pH neutral counterparts (Gʹ∼3000-4000 Pa), consistent with more extensive early SF/PP network formation under acidic conditions[Fig. 2(C & D)]. At low applied stress, G′ remained greater than G″ for both formulations, indicating predominantly elastic/solid-like behaviour. With increasing stress, G′ decreased and eventually crossed G″, indicating transition toward viscous flow. Importantly, after pH neutralisation, both formulations softened earlier and the G′-G″ crossover shifted to lower shear stress, resulting in a significantly lower flow point (<100 Pa) for each ink [**p < 0.0001,** Fig. 2(E)]. For extrusion printing, the flow point (G′-G″ crossover) is commonly used as a practical rheological characteristic for the onset of macroscopic flow, i.e., the stress level at which the material transitions from predominantly elastic/solid-like behaviour to a regime where it can flow through the nozzle. In the case of a silk-based bioink, this crossover is explicitly used to assess printability because it reflects whether applied shear can sufficiently disrupt/unfold/align the network to enable flow, while still enabling the ink to behave as a viscoelastic solid once shear is removed [29]. In this context, the higher flow points at acidic pH are consistent with our earlier observation of rapid, inhomogeneous gelation resulting in higher resistance to flow and greater risk of nozzle clogging [Fig. 1(B)]. The lower flow points after neutralisation rationalise the improved extrudability and handling window, requiring less stress (and therefore typically lower pressure) to initiate flow during extrusion, while still maintaining a solid-like regime at low stress prior to crossover that supports filament formation. The pH-neutralised formulations also exhibited reproducible structural recovery in 3ITT cyclic step-strain recovery test **[**Fig. 2(F–I)]. At low-strain condition (1%), both formulations showed a predominantly elastic response. Increasing the strain to 500% caused an abrupt reduction in G′ and complex viscosity, indicating shear-induced disruption of the physically cross-linked SF/PP network. However, returning the strain to 1% resulted in rapid recovery of G′ and complex viscosity, and interestingly, a comparable breakdown-recovery response was observed during the second high-strain cycle. Specifically, SF6PP12 showed recovery close to its initial low-strain state, whereas SF6PP26 exhibited a somewhat lower recovered modulus and complex viscosity, consistent with its overall softer rheological response. This reversible shear-induced network disruption and subsequent restructuring is beneficial for extrusion printing, where the bioink must flow during passage through the nozzle and rapidly recover its viscoelastic structure after deposition. Collectively, these data confirm that pH neutralisation not only extends the sol-gel working window, but also yields inks with favourable shear-dependent flow expected to support stable extrusion and continuous filament deposition.

**Fig. 2:**
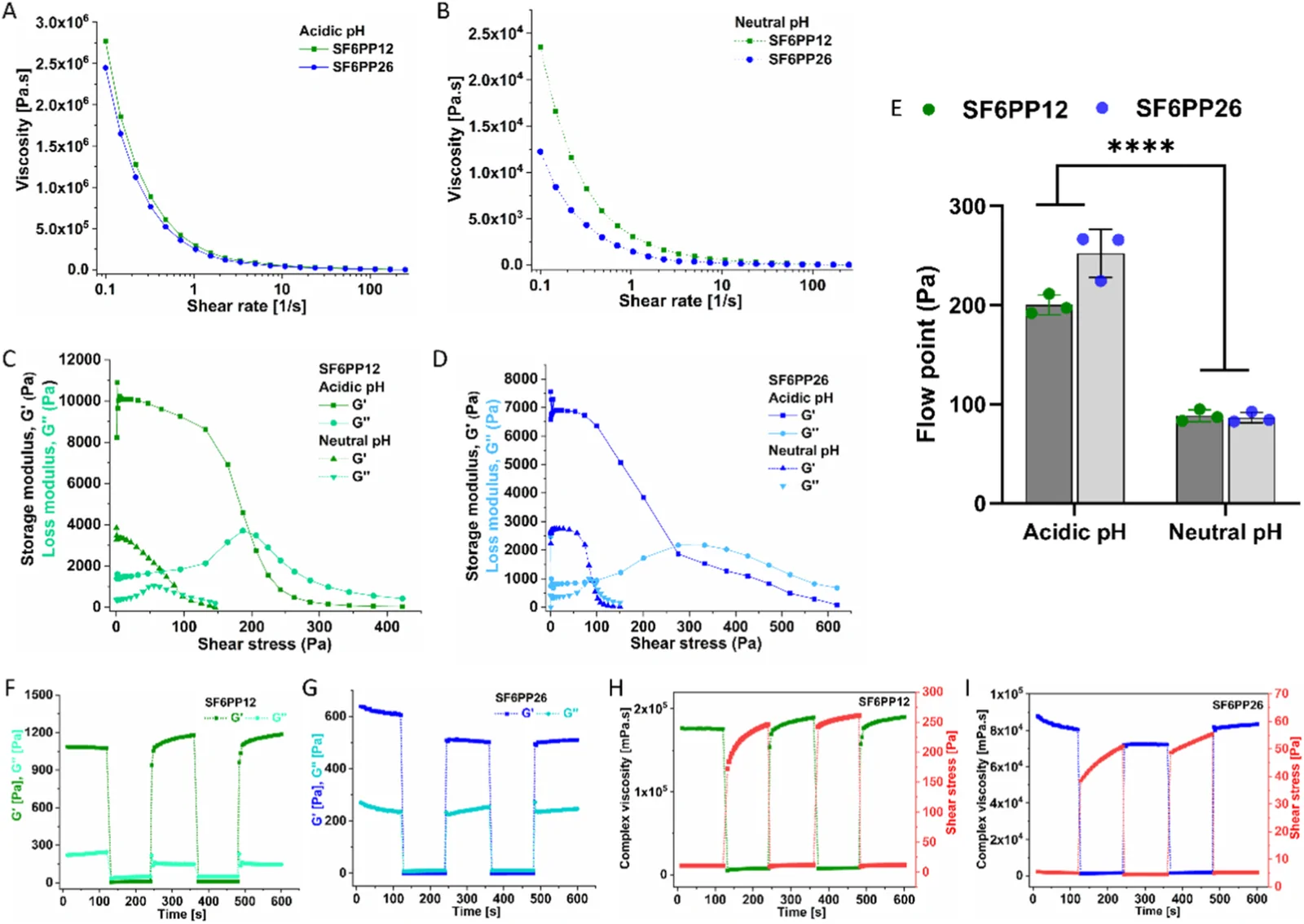
Rheological properties of SF/PP formulations after 15 min of gelation, showing shear-thinning behaviour under acidic (A) and pH neutralised (B) conditions**.** Separate viscosity y-axis ranges are used to allow the shear-thinning profiles under both conditions to be clearly resolved. (C-D) Oscillatory amplitude sweeps showing storage (G′) and loss (G″) moduli versus applied shear stress for SF6PP12 and SF6PP26 at acidic and neutral pH, demonstrating higher viscoelasticity and delayed yielding at acidic pH, and earlier yielding/softening after neutralisation. (E) Quantification of the flow point (derived from the amplitude sweep cross-over) comparing acidic vs neutral pH for both formulations. Thixotropic behaviour, showing (F & G) step-strain time sweep (strain recovery) demonstrating dynamic mechanical reversibility and resilience to cyclic deformation and (H & I) rapid recovery of complex viscosity post-deformation under alternating shear stress. In (E), data are Mean ± S.D. (n = 3), ****p < 0.0001 (Two way ANOVA).

Following rheological confirmation that pH neutralisation delayed premature SF/PEDOT network consolidation and preserved an extrusion-compatible working window, the printability of the neutralised SF6PP12 and SF6PP26 bioinks was quantitatively assessed using a CELLINK BIO X™ pneumatic extrusion bioprinter. Preliminary formulation screening showed that a lower 3 wt% SF formulation remained extrudable but lacked sufficient self-standing filament stability [Fig. S6]; therefore, 6 wt% SF was carried forward for quantitative printability assessment. Because the two formulations exhibited different viscosity-dependent rheological behaviours, their printability was evaluated within two pressure ranges using a 22G nozzle: 45-55 kPa and 56-70 kPa. The uniformity factors remained close to unity, with values of 1.133 ± 0.052 and 1.079 ± 0.057 for SF6PP12, and 1.029 ± 0.027 and 1.140 ± 0.031 for SF6PP26 at 45-55 and 56-70 kPa, respectively (mean ± SD, n = 5), indicating generally uniform filament extrusion [Fig. 3(A)] [32]. Spreading ratio (SR) values were greater than 1 for both formulations, indicating lateral filament spreading after deposition [Fig. 3(B)]. This is expected for hydrated hydrogel inks, where polymer-chain relaxation and residual viscous flow after extrusion led to strand widths exceeding the nozzle-defined dimension. The higher-pressure window generally increased spreading, consistent with pressure-driven increases in material flow and over-deposition. SF6PP26 showed a stronger tendency toward spreading than SF6PP12, indicating that the higher PEDOT-containing formulation remains printable but requires tighter pressure control to preserve filament definition. This behaviour aligns with the dynamic printability-window concept, where extrusion pressure, filament spreading, and pore fidelity collectively define the useable processing space rather than any single printability metric alone [32]. Despite this spreading, printability index (Pr) values remained close to 1, demonstrating preservation of square-like pore geometry in printed grids [Fig. 3 (C& F)]. Since Pr ∼1 corresponds to near-ideal square pores, these results show that both SF6PP12 and SF6PP26 can maintain construct-level lattice fidelity under extrusion printing. The representative grids further support this interpretation, showing open pores and continuous filament paths without gross pore closure or collapse [Fig. 3(F)]. Therefore, while SF6PP26 shows greater pressure-sensitive spreading at the filament scale, its overall pore architecture remains within an acceptable printability window.

**Fig. 3:**
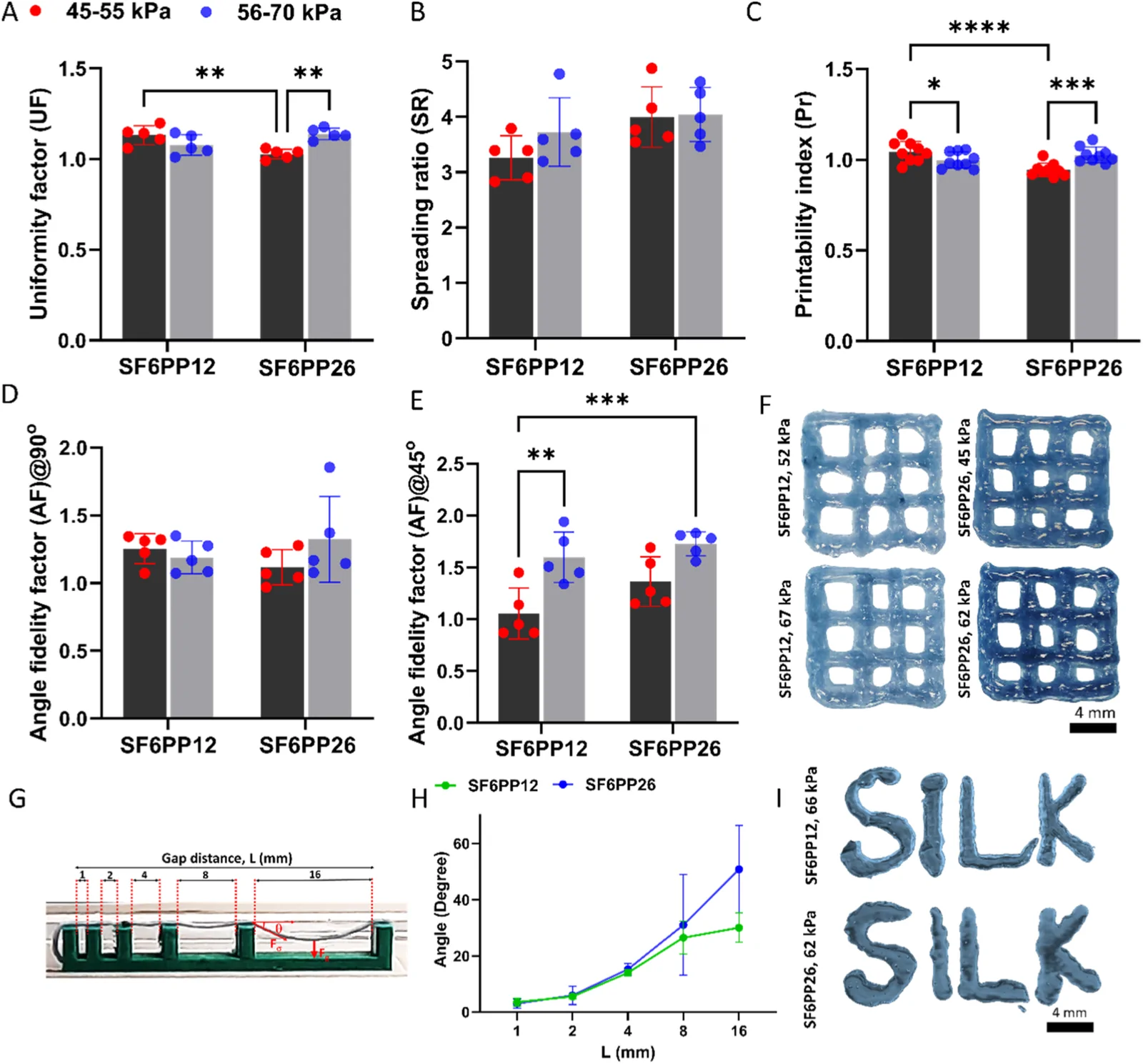
**Extrusion based printability and shape-fidelity assessment of pH-neutralised SF/PP bioinks.** (A-C) Quantitative evaluation of printing metrics showing (A)uniformity factor (UF), (B) spreading ratio (SR), and (C) printability index (Pr) for SF6PP12 and SF6PP26. (D,E) Angle-fidelity analysis showing pressure-dependent corner accuracy and material accumulation when printed at (D) 90° and (E) 45° geometries. (F) Representative printed grid constructs showing pore retention and macroscopic shape fidelity of SF6PP12 and SF6PP26, used for Pr index measurement. (G) Representative filament-collapse test setup used to evaluate unsupported filament stability across increasing gap distances and (H) filament deflection angle as a function of gap distance. (I) Representative freeform “SILK” constructs printed from SF6PP12 and SF6PP26, confirming the ability of the optimised formulations to retain continuous filament deposition and construct-level geometry. Data are presented as mean ± SD, n = 5 or more (*p < 0.05, **p < 0.01, ***p < 0.001, and ****p < 0.0001; Two-way ANOVA).

Angle fidelity analysis further distinguished pressure-sensitive geometric deformation between the two formulations [32]. At 90°, both bioinks retained relatively stable corner definition [Fig. 3(D)], whereas the 45° geometry provided a more stringent test of shape fidelity [Fig. 3(E)]. The sharper angle amplified vertex accumulation and corner thickening, particularly in the higher-pressure window. This effect was more apparent for SF6PP26, consistent with its higher SR and greater tendency toward material spreading. Thus, acute-angle geometries reveal the upper boundary of the printable pressure range more clearly than straight-filament or square-pore measurements alone. For SF/PP bioinks, the lower extrusion pressure window is therefore more suitable, particularly for SF6PP26, when fine angular features or high-resolution patterns are required. Representative printed constructs confirmed that the optimised printing conditions supported both grid-like [Fig. 3(F), **Movie S1-3,** Supporting **Information**] and freeform architectures [Fig. 3(I), **Movie S4**, Supporting Information]. SF6PP12 produced slightly sharper strand boundaries and more stable pore definition, whereas SF6PP26 remained printable but showed visibly thicker filaments and greater edge broadening. This formulation-dependent difference reflects the central material trade-off and suggests that increasing PEDOT content improves the electroconductive character of the bioink, but also shifts the formulation closer to the spreading-dominated boundary of the printability window owing to their lower viscosity [Fig. 2(B)].

Filament-collapse testing was then used to assess unsupported filament stability across increasing gap distances of 1, 2, 4, 8, and 16 mm [Fig. 3 (G & H)]. This test complements pore-based analysis as it probes gravitational sagging of suspended strands rather than only x-y plane shape fidelity [33]. Both bioink formulations showed low deflection across short spans, indicating adequate self-support after deposition. As the unsupported gap increased, the deflection angle (θ) progressively increased, confirming span-dependent sagging under filament mass. SF6PP26 (45-50 kPa) displayed greater long-span deflection than SF6PP12 (52-55 kPa), particularly at the larger gap distances, indicating lower resistance to unsupported collapse under the optimised printing windows.

The collapse behaviour is consistent with the filament-level metrics. SF6PP12 showed better resistance to spreading and sagging, whereas SF6PP26 preserved printability but with a narrower processing tolerance. This difference is important since SF6PP26 is the more bioelectronically relevant formulation due to its higher PEDOT content, yet its geometry is more sensitive to pressure. Specifically, SF6PP26 exhibited improved printability at lower extrusion pressure, a desirable feature for cell-laden bioprinting because it can reduce shear stress exposure and help preserve cell viability.

Overall, these findings are consistent with previous silk-based bioink studies showing that silk concentration and network formation strongly influence extrusion fidelity. Low-concentration silk systems typically show limited shape retention, whereas increasing silk content or promoting additive-mediated gelation improves filament formation and construct stability [41]. For example, Zheng et al. reported that SF/polyethylene glycol (PEG) bioinks containing 5-10 wt% silk exhibited improved viscosity, storage modulus and shape fidelity compared with lower silk concentrations of ∼2-3.5 wt%, with the 7.5 wt% formulation supporting stable multilayer printing and long-term construct integrity in biologically relevant environments. As mentioned above, in the present SF/PP system, 3 wt% SF enabled filament extrusion but produced constructs that were too soft to maintain self-standing fidelity [Fig. S6], whereas higher silk concentrations increased viscosity and promoted nozzle clogging. Together with the rheological and printability assessments, these observations identify 6 wt% SF as an optimal concentration for balancing extrusion, filament stability and conductive bioink functionality in the SF/PP hydrogel system.

### 3.2 PEDOTGoP-mediated network remodelling tunes SF/PP hydrogel mechanics, swelling/structural stability and hiCM compatibility

Having established printability of pH-neutralised SF/PP formulation, we next investigated how PEDOT*GoP* incorporation and short-term aqueous equilibration modulated mechanics, swelling behaviour and microstructure of the resulting hydrogels. This is a key design consideration for cardiac bioelectronic interfaces, where the material must retain sufficient integrity for handling and shape maintenance while presenting a hydrated matrix that can support hiCM attachment and early adaptation. Representative compressive stress-strain curves revealed clear formulation and incubation-dependent changes in hydrogel mechanics [Fig. 4(A)]. Before incubation, SF6 displayed the highest resistance to compression, whereas incorporation of PEDOT:PSS reduced the compressive modulus [Fig. 4(B)]. This decrease became more pronounced after 24 h incubation in PBS at 37°C, where both SF6PP12 and SF6PP26 softened substantially compared with pure SF6 (SF6 *vs* SF6PP12, p < 0.01; SF6 *vs* SF6PP26, p < 0.05). A similar trend was confirmed by oscillatory rheology, with the storage modulus decreasing after aqueous incubation and showing lower values in the PEDOT-containing formulations, particularly SF6PP26 [Fig. 4(C), p < 0.0001]. These results indicate that PEDOT*GoP* incorporation modifies the SF network rather than simply acting as a passive conductive filler. The reduced modulus was accompanied by increased swelling and a more open pore architecture. Lyophilised SF6PP12 and SF6PP26 scaffolds showed higher mass-swelling ratios than SF6, both immediately after gelation and after PBS preconditioning [Fig. 4(D)]. SEM imaging revealed clear formulation-dependent differences in the microarchitecture of the lyophilised scaffolds [Fig. 4(E)]. Bulk porosity was quantified using the ethanol-displacement method [Fig. 4(F)]. SF6 showed a markedly lower porosity (∼11%) than SF6PP12 (∼50%) and SF6PP26 (∼52%), with both SF/PP formulations exhibiting significantly higher porosity than SF6 (**p < 0.01 and ***p < 0.001, respectively)**.** Together, these results suggest that PEDOT*GoP* disrupts dense silk-silk packing and increases fluid-accessible free volume, leading to lower bulk resistance to compression after PBS incubation.

**Fig. 4:**
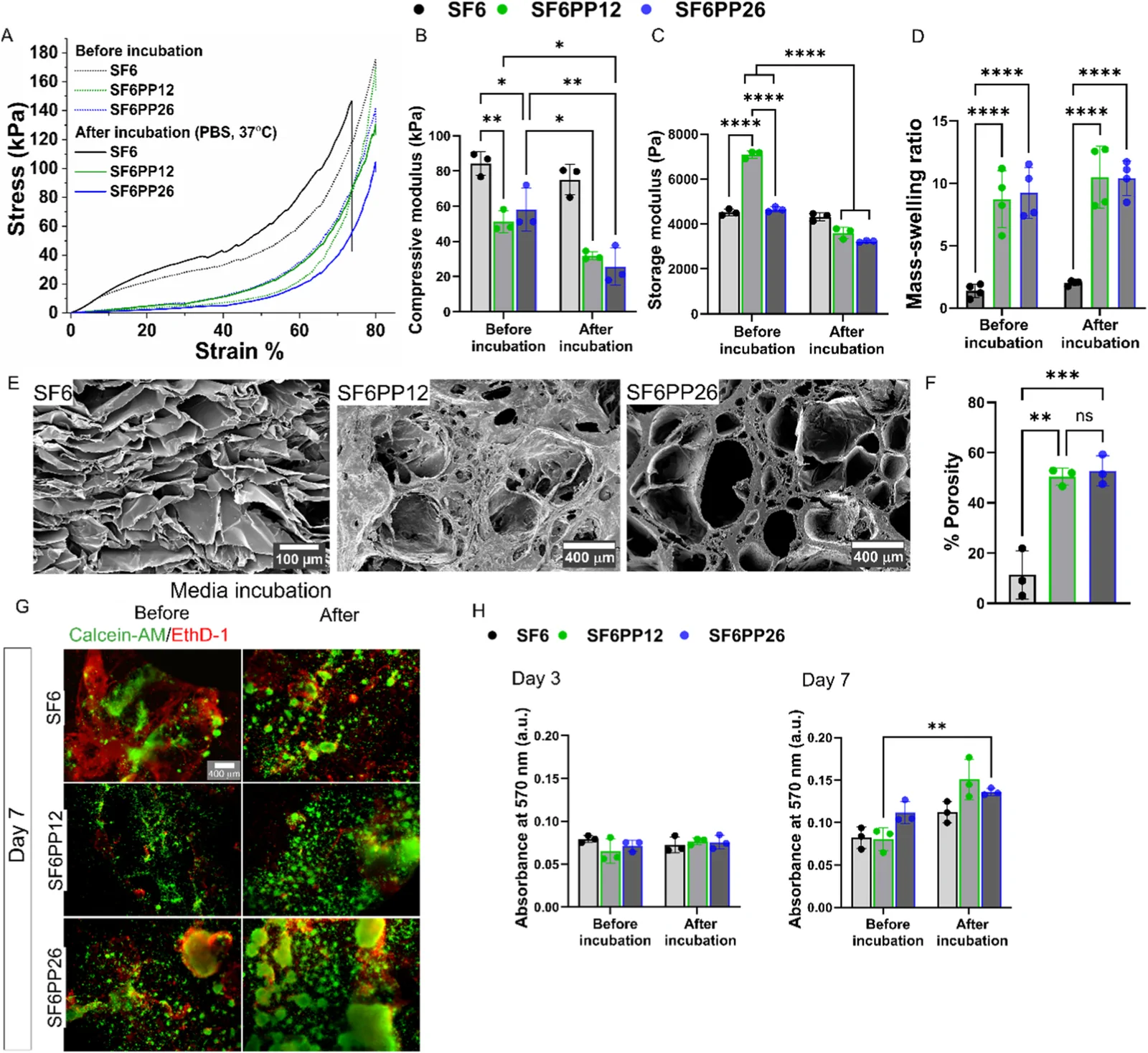
**PEDOT:PSS-mediated network remodelling regulates the mechanics, swelling, porous architecture, and early hiPSC-cardiomyocyte compatibility of SF/PP hydrogel constructs.** (A) Representative compressive stress-strain curves of SF6, SF6PP12, and SF6PP26 hydrogels before and after 24 h aqueous incubation at 37°C and their derived (B) compressive modulus. (C) Storage modulus of the hydrogel formulations before and after incubation. (D) Mass-swelling ratio of lyophilised scaffold networks prepared immediately after gelation or following 24 h incubation. (E) Representative SEM images of lyophilised SF6, SF6PP12, and SF6PP26 scaffolds [Scale bars: 100 μm (SF6) and 400 μm (SF/PP)] and (F) Bulk porosity of SF6, SF6PP12 and SF6PP26 scaffolds quantified using the ethanol-displacement method. (G) Live/dead staining of hiPSC-derived cardiomyocytes cultured on freshly prepared and media-preconditioned hydrogels at day 7 showing viable cells in green (Calcein-AM) and dead in red (EthD-1). [Scale bar: 400 μm]. (H) MTT metabolic activity at days 3 and 7, showing comparable early activity at day 3 and enhanced day-7 activity on preconditioned hydrogels. Data are presented as mean ± SD. Statistical significance is indicated as *p < 0.05, **p < 0.01, ***p < 0.001, and ****p < 0.0001.

The mechanical range achieved here is relevant for cardiac biointerfaces. Commonly used cardiac mechanobiology benchmarks place healthy myocardium at ∼8-15 kPa, whereas infarcted/fibrotic myocardium is typically reported in the ∼20-100 kPa range, with mature post-MI scar often reported around ∼35-70 kPa [42]. Within this context, the SF/PP hydrogels in their freshly formed state (∼40-60 kPa) fall within a scar-like mechanical regime, while 24 h of PBS incubation softens them to ∼20-30 kPa, shifting them toward the lower end of the remodelled/fibrotic range. Nevertheless, comparable mechanical ranges, as well as substantially stiffer but structurally compliant materials, have been employed in cardiac biomaterials and bioelectronic interfaces. For example, Ryu et al. reported a tissue-adhesive hydrogel with a Young's modulus of ∼30 kPa for an electrically conductive cardiac patch [43], while Fischer et al. demonstrated long-term hiCM culture for up to 60 days on 3D printed alginate scaffolds with a Young's modulus ∼20 kPa [44]. In a distinct cardiac bioelectronic design, Asulin et al. integrated a substantially stiffer graphite/PDMS electrodes with a Young's modulus of ∼1 MPa into a 3D-printed cardiac patch [17]. They showed that despite the substantially higher material stiffness, the serpentine architecture provided sufficient mechanical compliance to support deformation, cardiac-cell culture, electrical recording and pacing. Although these values were obtained using different mechanical testing modalities and are therefore not directly equivalent to the compressive modulus of our SF/PP hydrogels, they provide relevant precedent for cardiac interfacing and cardiomyocyte culture within a similar stiffness range. Short-term aqueous equilibration relaxes and plasticises the physically crosslinked SF/PP network, increasing fluid-filled free volume and reducing bulk resistance to deformation, which may be advantageous for accommodating the dynamic mechanical environment of beating cardiomyocytes and cardiac tissue. This interpretation is consistent with classic silk hydrogel studies showing that pore architecture is closely linked to mechanical behaviour, where denser silk networks with smaller pores exhibit higher compressive strength and modulus, while more open structures are mechanically softer [22]. In addition, these SF/PP bioink formulations are biodegradable as evident from protease XIV-mediated mass loss over 30 days, suggesting their progressive network remodelling features under physiological condition [Fig. S7, Supporting Information]. Such progressive network remodelling further suggests that the initial ∼20-30 kPa modulus may evolve over time, potentially supporting improved long-term mechanical adaptation at the healthy myocardium interface.

Live/dead staining at day 7 showed that all hydrogel formulations supported hiCM survival [Fig. 4(G)]. However, preconditioned hydrogels showed denser Calcein-AM + ve cell coverage than freshly prepared hydrogels, particularly in SF6PP12 and SF6PP26. Representative phalloidin-stained images of the media-preconditioned SF6PP12 and SF6PP26 groups further showed adherent cells with F-actin-positive cytoskeletal spreading and bundles, along with localised cell clustering, typically observed for hiPSC derived cardiomyocytes [Fig. S8]. The MTT assay showed comparable metabolic activity across groups at day 3, indicating no acute cytotoxicity [Fig. 4(H)]. By day 7, the cells on the SF/PP hydrogels showed considerable metabolic activity, with relatively higher activity in the preconditioned groups, suggesting no cytotoxic effects of these materials. These results suggest that short-term preconditioning improves the early cell-biomaterial interactions, likely through coupled changes in hydration, stiffness and pore architecture. Overall, PEDOT*GoP* incorporation remodelled the SF hydrogel network by increasing swelling capacity and porosity while reducing compressive and storage moduli. This mechanics-swelling-microstructure transition provides a material basis for the improved day 7 hiCM coverage and metabolic activity observed on preconditioned SF/PP hydrogels. Thus, SF6PP12 and SF6PP26 provide a suitable balance of structural stability, hydration, compliance and cytocompatibility for subsequent cardiac bioelectronic interfacing studies.

### 3.3 Dynamic mechanical stability and interfacial integrity of printed SF/PP constructs

Since a cardiac interface would be exposed to repetitive mechanical deformation associated with the beating heart, we further evaluated the dynamic mechanical stability of the printed SF/PP constructs using unconfined cyclic compression at 25% strain and 1 Hz, as a cardiac-cycle-relevant loading condition, upto 50 cycles [Fig. 5]. As compared to complex multiaxial deformation of native myocardium, unconfined compression of soft hydrogels results in coupled deformation including both axial compression accompanied by lateral expansion and tensile deformation of the polymer network [45].

**Fig. 5:**
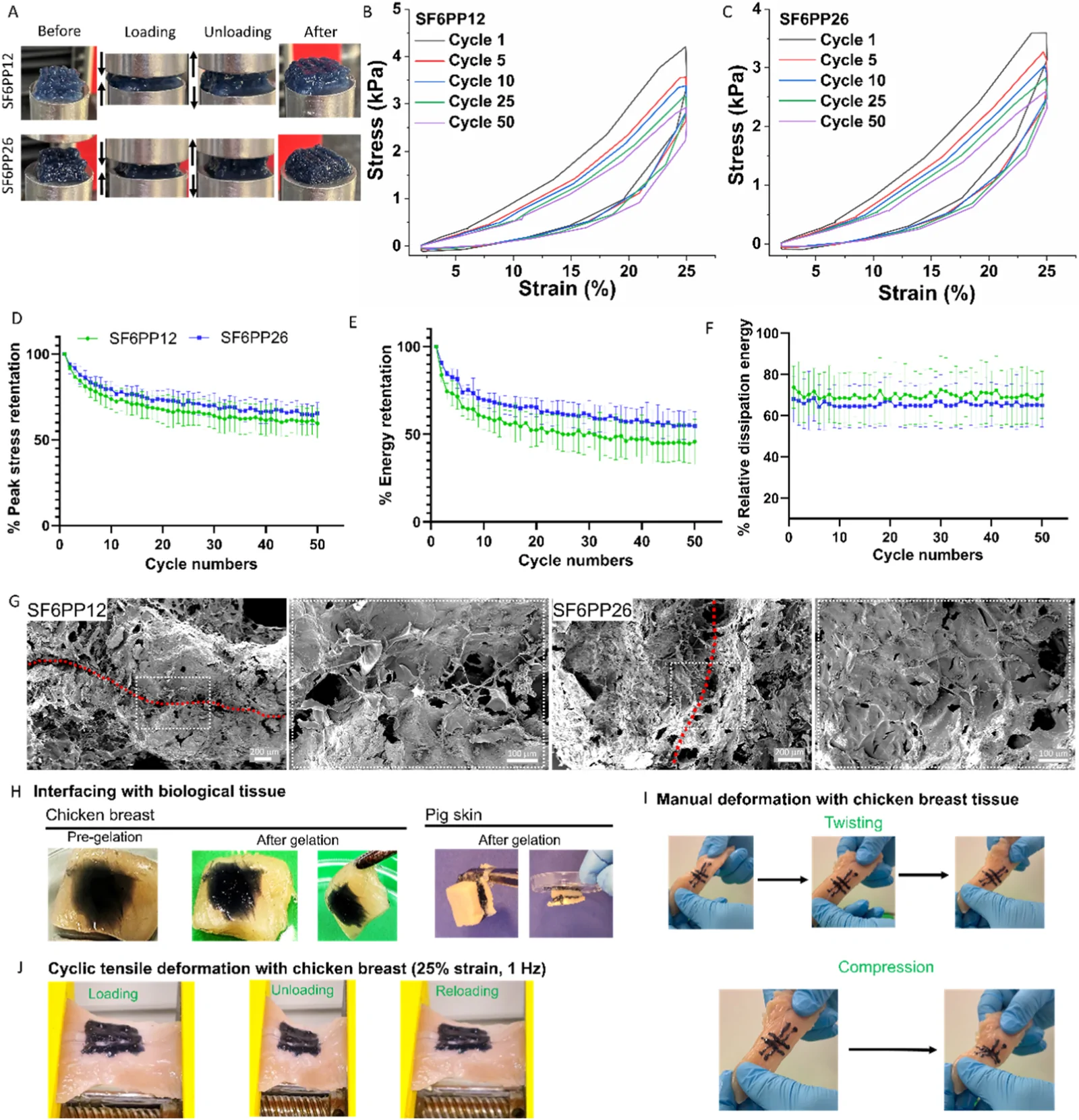
**Dynamic mechanical stability, interfilament integrity and qualitative tissue adherence of printed SF/PP constructs.** (A) Representative SF6PP12 and SF6PP26 constructs before, during and after cyclic compression. (B,C) Stress-strain hysteresis curves at cycles 1, 5, 10, 25 and 50 during unconfined cyclic compression at 25% strain and 1 Hz. Quantification of (D) peak-stress retention, (E) dissipated-energy retention and (F) relative energy dissipation over 50 cycles (mean ± SD, n = 3). (G) Cross-sectional SEM of printed constructs showing coalesced filament-filament interfaces; red dashed lines indicate nominal interfilament boundaries and boxed regions are shown at higher magnification. (H) Representative images of SF/PP hydrogel retention on ex-vivo chicken breast tissue and pig skin before or after gelation as labelled. (I) Visual macroscopic stability of manually extruded SF/PP filaments on chicken breast tissue during twisting and compression. (J) Representative loading-unloading-reloading sequence during cyclic tensile deformation of the hydrogel-tissue interface at 25% strain and 1 Hz.

Printed SF6PP12 and SF6PP26 constructs, at representative cycles 1, 5, 10, 25 and 50, exhibited pronounced hysteresis together with a progressive reduction in maximum stress and loop area [Fig. 5(B & C)], suggesting a viscoelastic and stress-softening response rather than purely elastic deformation. The results showed peak stress retention of 81.2 ± 3.8% and 86.3 ± 4.1% at cycle 5, which further decreased to 59.5 ± 8.6% and 65.5 ± 6.1% by cycle 50 for SF6PP12 and SF6PP26, respectively [Fig. 5(D)]. The hydrogel constructs also showed reduction in dissipated-energy retention (hysteresis energy dissipated in each later cycle relative to that dissipated in cycle 1). For SF6PP12 and SF6PP26, dissipated-energy retention decreased from 71.5 ± 7.4% and 81.6 ± 5.4% at cycle 5 to 45.7 ± 12.8% and 54.6 ± 8.2% at cycle 50, respectively [Fig. 5(E)]. This reduction indicates that the absolute hysteresis-loop area became progressively smaller as the constructs underwent repeated loading, consistent with cyclic stress softening and mechanical conditioning of the hydrated network. Interestingly, SF6PP26 displayed numerically greater retention of both peak stress and dissipated energy throughout the test, suggesting a somewhat more persistent mechanical response under repeated compression. Importantly, the gradual reduction in these parameters was not accompanied by an abrupt loss of load-bearing capacity or visible macroscopic failure, and both printed constructs remained structurally intact throughout the 50 cycle test [Fig. 5(A) and Movie S14].

Despite this progressive reduction in absolute stress and dissipated energy, the relative energy dissipation (hysteresis energy normalised to the loading energy within each cycle), remained comparatively stable [Fig. 5(F)]. SF6PP12 exhibited relative dissipation of 73.8 ± 10.3% at cycle 1 and 70.1 ± 11.5% at cycle 50, while SF6PP26 changed only from 68.0 ± 11.2% to 64.9 ± 10.5% over the same period. The reduction in dissipated-energy retention reflects the decreasing absolute mechanical work associated with progressive softening, whereas the nearly conserved relative dissipation indicates that the conditioned constructs continued to dissipate a similar fraction of the mechanical energy imposed during each loading cycle.

The observed hysteresis and early-cycle stress softening are typical features of hydrated polymer networks like in hydrogel, which are attributed to progressive mechanical conditioning, stress relaxation and structural/fluid rearrangement [30], [31]. Therefore, the progressive stress softening in the physically crosslinked SF/PP system, likely reflects repeated mechanical conditioning of the hydrated SF/PP network, involving water redistribution and reversible rearrangement of polymer chains and physical interactions during cyclic deformation. The overall structural integrity of the constructs during the cyclic compression remained preserved as evident macroscopically without visible tearing, delamination or collapse after cyclic compression [Fig. 5(A)]. This macroscopic stability was further supported by cross-sectional SEM of the printed constructs [Fig. 5(G)], where adjacent filaments displayed continuous, coalesced contact regions without obvious open gaps or interfacial separation.

Interfacing ability of the SF/PP hydrogels with biological tissue along with qualitative mechanical stability was also evaluated [Fig. 5(H–J)]. SF6PP26 hydrogel was deposited onto chicken breast tissue and allowed to gel for 30 min at 37°C, which remained visibly associated with the tissue during subsequent handling; similar macroscopic retention was observed after gelation on pig skin [Fig. 5(H)]. Manually extruded SF6PP26 filaments on chicken breast tissue also retained their overall attachment and printed pattern during twisting and compression of the underlying tissue [Fig. 5(I)]. To evaluate tissue-interface stability under a more controlled dynamic deformation, SF/PP hydrogel was deposited onto chicken breast tissue mounted in a custom-built, Arduino-controlled electromechanical system and subjected to cyclic tensile deformation at 25% strain and 1 Hz. Representative loading-unloading-reloading frames (with SF6PP26) showed that the hydrogel remained macroscopically associated with the deforming tissue surface without visual detachment macroscopically [Fig. 5(J); **Movie S15-16**]. Overall, these observations provide preliminary qualitative evidence that the SF/PP hydrogels can maintain tissue-surface contact during both manual deformation and under cardiac-cycle-relevant dynamic loading.

### 3.4 Bioprinting of hiPSC derived cardiac fibroblasts

Having established the extrusion printability of the SF/PP formulations, we next assessed their ability to support cell-laden processing using hiCFs as a cardiac-relevant stromal cell model. Cardiac fibroblasts represent a major stromal population within the myocardium and play central roles in extracellular-matrix organisation and remodelling [35]. Furthermore, cardiomyocytes have been reported to exhibit greater sensitivity than cardiac fibroblasts to extrusion pressure and bioink processing conditions [46]. Accordingly, hiCFs were used here as a proof-of-concept model to evaluate cell incorporation, extrusion and short-term post-printing cytocompatibility of the optimised SF/PP formulations, rather than as a functional 3D myocardial model. hiCFs were encapsulated within the SF/PP bioinks and successfully extruded into both tubular and grid-like constructs [Fig. 6(A and B]. Both formulations produced continuous cell-laden filaments and retained the intended macroscopic geometry after printing, confirming that the pH-neutralised SF/PP system preserved sufficient extrudability and post-deposition shape stability for simple 3D biofabrication. Live/dead staining showed that hiCFs remained predominantly viable within both SF6PP12 and SF6PP26 constructs over the 96 h post-printing period [Fig. 6(B)]. In both tubular and grid shaped constructs, Calcein-AM-positive cells were distributed throughout the printed regions, with comparatively limited EthD-1-positive signal. Quantification of the Calcein-AM + ve area fraction, normalised to the printed area, showed a significant increase in viable-cell coverage from 12 to 24 h in SF6PP12, which was maintained at 96 h [Fig. 6(C)]. SF6PP26 showed slightly higher Calcein-AM + ve cell coverage than SF6PP12 across all time points, although this difference was not statistically significant.

**Fig. 6:**
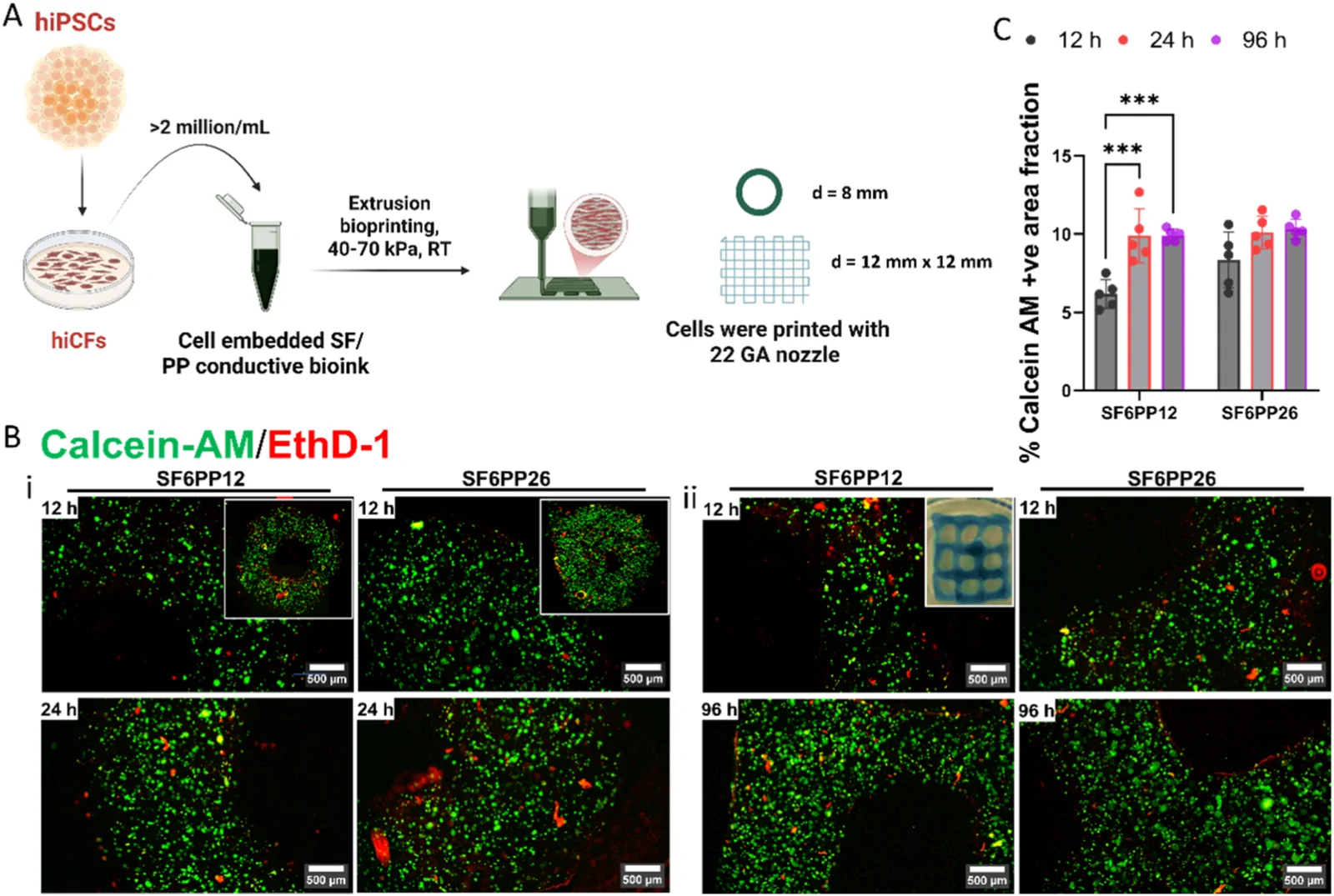
**Extrusion bioprinting of hiPSC-derived cardiac fibroblast (hiCFs)-laden SF/PP conductive bioinks. (A)** Schematic illustration of the bioprinting workflow. hiCFs were embedded in SF/PP conductive bioinks and extruded through a 22 G nozzle at room temperature to fabricate circular/tubular and grid-shaped constructs. **(B)** Representative live/dead fluorescence images of hiCF-laden (i) tubular and (ii) grid-shaped SF6PP12 and SF6PP26 printed constructs at 12, 24 and 96 h after bioprinting, as labelled. **(C)** Quantification of hiCF viability within SF6PP12 and SF6PP26 printed constructs at 12, 24 and 96 h after bioprinting, represented as the percentage of Calcein-AM-positive area fraction normalised to the printed area. Insets in B(i) show reconstructed fluorescence images of full tubular cell-laden constructs, while the inset in **B(ii)** shows a representative digital image of a cell-laden grid-shaped construct. Scale bars: 500 μm. Data are presented as mean ± SD. ***p < 0.001.

Previous studies showed increasing silk content increases stiffness, which in turn reduces cell survival [47]. However, stiffness and cell survival are also formulation dependent. For example, pure SF6 had high stiffness and reduced porosity as compared to SF/PP formulations as shown in Fig. 4; which in turn showed reduced cell survival in SF6 hydrogel (not printed) over time [Fig. S9]. Although nutrient mass transport was not directly quantified, the lower porosity and denser network of SF6 may have contributed to a comparatively less permissive cellular microenvironment. In contrast, the addition of PEDOT*GoP* mediated gelation alters network hydration and microstructure [Fig. 4], which ultimately improved cell viability more so than pure silk hydrogels at the same concentration. This is consistent with earlier studies reporting long-term stem cell viability in SF/PEG hydrogels with 7.5 wt% silk content [41] and 6 wt% riboflavin-crosslinked SF hydrogels [48]. Collectively, these findings establish short-term cell-laden extrusion feasibility of the SF/PP formulations using a cardiac-relevant stromal cell model, while further cell-specific optimisation would be required to extend the platform to direct 3D hiCM-laden printing.

### 3.5 Electroactivity and stiffness regulate hiPSC cardiomyocytes contractility and structural organisation

After establishing proof-of-concept cell-laden extrusion with hiCFs, we next explore the functional bioelectronic interfacing of the platform by evaluating hiCMs cultured at the conductive SF/PP interface during capacitive-coupling WES. Restoring bioelectrical signalling after myocardial injury is essential to maintain normal electromechanical functioning of the heart. In this context, an electroconductive cardiac patch is an ideal choice to promote electrical impulse propagation in an infarcted region, which can also be used to deliver exogenous ES. Multiple studies have shown that conductive hydrogels can support electrical signalling and improve post-infarct cardiac function [15], while electrical pacing enhances force generation and maturation of hiPSC-derived cardiac tissues [49]. More recently, PEDOT:PSS-containing cardiac hydrogels were shown to improve hiPSC-cardiomyocyte contractility, calcium handling, and conduction velocity while reducing post-infarct ventricular tachycardia, further highlighting the therapeutic relevance of electroactive soft interfaces for myocardial repair [16].

Fitting of electrochemical impedance spectroscopy (EIS) measurements in PBS demonstrated a decrease in charge-transfer resistance from 834 ± 16 Ω cm^2^ for SF6PP12 to 424 ± 12 Ω cm^2^ for SF6PP26 [Fig. 7(A and B); Figure S11(C&D)]. This indicates more efficient interfacial charge transport with increasing PEDOT*GoP* concentration [Fig. 7(A and B)]. Bode plots show a lower impedance across the entire frequency range for SF6PP26 [Figure S11(A&B)], confirming higher conductivity with increasing PEDOT*GoP* concentration. In both cases, the magnitude of the phase is small at high frequencies, implying resistive behaviour, as the frequency decreases, the phase increases in magnitude, approaching capacitive behaviour (−90°) at low frequencies. SF6PP26 crosses −45° phase at around ∼32000 Hz, much higher than the ∼15000 Hz of SF6PP12. This implies a smaller time constant and therefore smaller charge transfer resistance, as was seen in the fitting of the Nyquist plots [Fig. 7(A and B); Figure S 11(C&D)]. EIS confirms the charge transfer behaviour of these conductive hydrogel bioink formulations in physiologically relevant ionic environment, which is particularly important while interfacing with electroactive biological tissue.

**Fig. 7:**
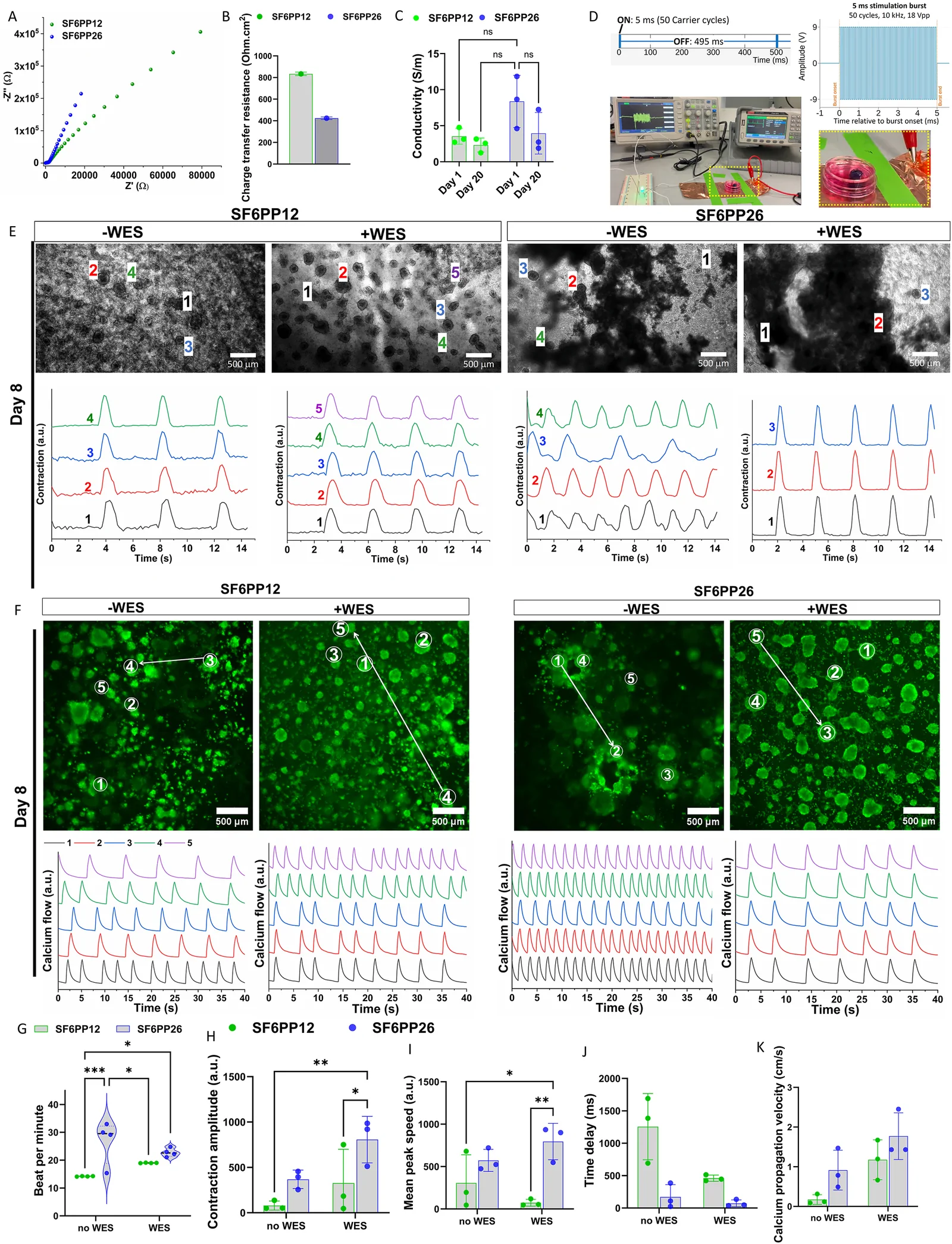
**Electroconductive SF/PP hydrogel bioinks support capacitive wireless electrical stimulation and regulate hiPSC-derived cardiomyocyte contractility and calcium handling.** (A) Nyquist plots and (B) charge-transfer resistance of SF6PP12 and SF6PP26 hydrogels measured in PBS. (C) Wet-state electrical conductivity measured on day 1 and after 20 days of PBS incubation at 37°C. (D) Representative waveform and benchtop setup for capacitive-coupling wireless electrical stimulation (WES), in which a 10 kHz biphasic carrier signal was delivered in 5 ms bursts at 2 Hz through a dielectric barrier to the conductive hydrogel receiver; LED illumination demonstrates signal transfer through the setup (**Movie S5,** Supporting Information). (E) Representative bright-field images of spontaneously beating hiPSC-derived cardiomyocytes cultured on SF6PP12 and SF6PP26 hydrogels with or without WES, with highlighted ROIs used for motion analysis using MUSCLEMOTION (**Movie S6-9,** Supporting Information) and the corresponding contraction traces. (F) Representative Cal-520 calcium imaging fields with highlighted ROIs (**Movie S10-13,** Supporting Information) and corresponding calcium transient profiles used to assess inter-ROI synchrony, time delay, and calcium wave propagation; white arrows indicate the spatial direction used for calcium propagation analysis. Quantification of contractile behaviour showing **(**G) beat rate quantified from contraction analysis, (H) relative contraction amplitude, and (I) mean peak contraction speed. Quantitative analysis showing (J) calcium transient time delay and (K) propagation velocity between highlighted ROIs with the white arrow. [Scale bar = 500 μm (E & F)]. Data are presented as mean ± SD, n = 3 or more. *p < 0.05, **p < 0.01, and ***p < 0.001.

In the hydrated state, this conductivity is expected to reflect mixed ionic–electronic transport, with ionic conduction through the aqueous phase occurring alongside electronic transport through the PEDOT:PSS network. Notably, freeze-dried SF/PP scaffolds retained measurable conductivity (∼0.005-0.02 S/m, Fig. S12), supporting an intrinsic PEDOT:PSS-mediated electronic contribution independent of the enhanced ionic transport present under hydrated conditions. Following 20 days of incubation in PBS, SF6PP12 and SF6PP26 retained conductivities of 2.32 ± 0.98 and 3.95 ± 2.88 S/m, respectively, demonstrating preservation of conductive pathways during prolonged aqueous exposure despite a reduction in the mean conductivity.

This reduction in conductivity was accompanied by gradual PEDOT:PSS-equivalent release, which remained comparatively limited under PBS conditions, reaching 96 ± 82 μg/mL for SF6PP12 and 82 ± 99 μg/mL for SF6PP26 at day 20 [Fig. S10]. In contrast, protease-mediated degradation resulted in a pronounced time-dependent increase in release to 463 ± 272 and 663 ± 343 μg/mL, respectively, indicating that mobilisation of PEDOTGoP is strongly associated with disruption of the surrounding SF network. The comparatively restrained release from the intact SF/PP network may additionally benefit from GOPS stabilisation, as GOPS crosslinking has been shown to suppress aqueous dissolution and delamination of PEDOT:PSS through interactions predominantly involving PSS-rich domains [40]. These findings suggest that the SF/PEDOT*GoP* network restricts rapid passive loss of the conductive phase while maintaining substantial wet-state conductivity over extended aqueous incubation.

To exploit this electroactivity, we implemented a non-invasive ES approach based on charge polarisation induced capacitive-coupling based power transfer following our previous work [13]. In this non-invasive ES system, a time-varying electrical signal is applied to a copper transmitter placed beneath the culture plate or glass bottom petri dish, while the polystyrene well base or the glass bottom acts as a dielectric barrier. The alternating field induced dielectric polarisation and displacement current across this barrier, generating a secondary electrical field within the conductive SF/PP hydrogel receiver. Because the hydrogel is a mixed ionic-electronic conductor, the induced field can polarise and mobilise internal charge carriers, thereby delivering an electrical cue to cardiomyocytes cultured on the hydrogel surface without direct electrode contact with the culture medium. This preserves the advantages of delivering wireless ES capacitively, while avoiding direct electrode-media electrochemical (faradaic) reactions and thereby, ensures biological safety.

For cardiomyocyte stimulation, a burst-modulated waveform was used rather than a continuous high-frequency signal. Specifically, a 10 kHz biphasic square-wave carrier with an amplitude of 18 Vpp (±9 V) was delivered in 5 ms bursts at 2 Hz as an arbitrary waveform [Fig. 7(D), detailed enlarged version shown in Fig. S13]. This design preserves efficient capacitive transfer in the kHz range, while presenting cardiomyocytes with an intermittent low-frequency stimulation envelope that is more relevant to cardiac excitation. Each 5 ms burst contains approximately 50 carrier cycles, enabling rapid capacitive transfer during the burst window without imposing continuous high-frequency stimulation. Under these conditions, the estimated electric field at the hydrogel interface was approximately 0.8-1.2 V/cm. The benchtop LED demonstration provides functional confirmation that burst packets can be transmitted through the capacitive setup to a conductive receiver, supporting the feasibility of this WES platform for cardiac bioelectronic stimulation [Fig. 7(D)–Movie S5, Supporting Information].

Given the high-frequency carrier used for capacitive coupling, we further evaluated whether the applied WES conditions generated appreciable Joule heating within the culture microenvironment. Temperature monitoring was performed using the same physical configuration used for WES of hiCMs, with SF/PP hydrogels deposited on the glass coverslip and immersed in RPMI culture medium but without cells. Four thermocouple probes were positioned within the culture setup, and temperature was monitored for SF6PP12 and SF6PP26 under WES and matched no-WES conditions for up to 48 h [Fig. S14(A&B)]. An increase in absolute temperature was observed during the initial period in both WES and no-WES conditions, reflecting equilibration of the culture setup within the incubator. Following equilibration, the temperature profiles remained relatively stable during the prolonged WES up to 48 h.

Since the thermocouple measurements are unable to identify a localised temperature increase within the conductive hydrogel or the immediately surrounding medium, we additionally performed electrothermal FEM using a three-dimensional representation of the experimental WES configuration [Fig. S14(C–E)]. An electrical conductivity of 3.5 S/m was assigned to the SF/PP hydrogel receiver. The predicted WES-associated temperature rise increased from 37°C (incubator temperature) to approximately 38°C (ΔT ≈ 1.2°C) within ∼4 h, with no further increase over the 48 h of simulation [Fig. S14(D)]. Spatial temperature distributions showed a broadly distributed thermal response with only a small temperature gradient across the culture medium, without a pronounced localised hotspot around the hydrogel [Fig. S14(E)]. The plateau in temperature profile is consistent with the intermittent stimulation waveform, where the 10 kHz carrier is delivered in 5 ms bursts at 2 Hz (1% duty cycle), limiting continuous energy deposition during prolonged stimulation. A similarly limited local electrothermal response was reported by Yang et al. during capacitive-coupling WES via PEDOT:PSS/PPy hydrogel at a higher carrier frequency of 5 MHz [12]. They also showed a modest surface temperature increase from 33.8 to 35.0°C over 30 min experimentally, while their FEM predicted <0.3°C heating around the implanted conductive hydrogel and adjacent tissue. Importantly, under their WES conditions, cell viability, proliferation and apoptosis/necrosis were not adversely affected. These findings are consistent with our experimental and computational results, indicating that the applied WES conditions produced minimal local Joule heating and that thermal effects are unlikely to account for the observed cellular responses.

hiPSC-derived cardiomyocytes were seeded on hydrogels with different stiffnesses after 24 h pre-incubation in RPMI medium and were subsequently subjected to the WES protocol. Bright-field videos of the cultures were recorded on day 8 and analysed using MUSCLEMOTION to assess contractile activity under non-stimulated and WES-treated conditions [Movie S6-9, Supporting **Information**]. Representative fields and ROI-specific MUSCLEMOTION traces in Fig. 7(E) show that cardiomyocytes cultured on both SF6PP12 and SF6PP26 hydrogels exhibited detectable spontaneous contraction profiles under both conditions. Qualitatively, the traces indicate repeated contraction events across multiple ROIs, confirming that both hydrogel formulations supported active beating behaviour. Following WES, the contraction traces appeared more regular across ROIs, although quantitative interpretation of functional differences was evaluated based on beat rate, relative contraction amplitude, and mean peak contraction speed, as shown in Fig. 7(G–I). Beat-rate analysis showed that cells beat relatively faster on softer and more conductive SF6PP26 (25 ± 9.26/minute) than on SF6PP12 (14.19 ± 0.10/minute) [Fig. 7(G), p < 0.001)]. Under ES, the beat rate on SF6PP12 increases to 19.01 ± 0.09, although there was no statistical significance. ES did not increase the beat rate on SF6PP26 but beating becomes more uniform across the multiple ROIs (22.07 ± 0.94). Relative contraction amplitude, which reflects the magnitude of contraction-associated motion per beat, showed higher mean values under WES in both hydrogel formulations. Two-way ANOVA revealed significant overall effects of WES (p = 0.0347) and hydrogel formulation (p = 0.0214) on contraction amplitude, although the within-formulation WES comparisons were not statistically significant (SF6PP12, p = 0.2310; SF6PP26, p = 0.0508). The strongest contraction response was observed in the SF6PP26 +WES group, with a statistically significant difference compared with SF6PP12 −WES [Fig. 7(H), p < 0.01]. Mean peak contraction speed, which reflects how rapidly contraction-associated motion develops within each beat, was significantly influenced by hydrogel formulation (p = 0.0035), whereas the overall WES effect was not statistically significant (p = 0.9607). Similarly, the within-formulation WES comparisons were not statistically significant for SF6PP12 (p = 0.2076) or SF6PP26 (p = 0.2301), although a significant difference was observed between SF6PP12 −WES and SF6PP26 +WES [Fig. 7(I), p < 0.05]. Collectively, these findings suggest that hydrogel formulation influences contraction-associated motion and kinetics, while WES was associated with an overall increase in contraction amplitude without significant differences within individual formulations. It should be noted that beating rate on these materials is far below 2 Hz burst-envelope frequency (i.e., equivalent to 120 beats/minute) of the applied ES. Therefore, WES was used here as a bioelectronic conditioning cue to modulate the contractility and structural organisation of the hiCMs.

Since cardiomyocyte contraction is intrinsically coupled to intracellular Ca^2+^ signalling through excitation-contraction coupling [50], we next examined whether the contraction profiles observed on SF/PP hydrogels were associated with coordinated Ca^2+^ signalling. ES has previously been shown to improve Ca^2+^ handling in stem-cell-derived cardiomyocytes, and conductive PEDOT:PSS based hydrogels have been reported to enhance bioelectrical coupling, Ca^2+^ propagation, and contractile function in engineered cardiac tissues [16]. Therefore, we performed live-cell Ca^2+^ imaging on day 8 using Cal-520 to assess intracellular Ca^2+^ transient behaviour in hiPSC-derived cardiomyocytes cultured on SF6PP12 and SF6PP26 hydrogels with or without capacitive WES. Live Ca^2+^ imaging videos [Movie S10-13, Supporting Information] and ROI-specific Ca^2+^ transient profiles [Fig. 7(F)] showed spontaneous Ca^2+^ activity across spatially separated ROIs in all groups, with differences in transient regularity and temporal coordination between the hydrogel formulations and stimulation conditions. The time delay and velocity of the Ca^2+^ wave propagation were measured between two distinct ROIs for all the groups [represented with white arrow in the fluorescent images in Fig. 7(F)]. Specifically, SF6PP12 exhibited a mean Ca^2+^ time delay of 1256 ± 512 ms without WES, which decreased to 461 ± 48 ms under WES, accompanied by an increase in apparent propagation velocity from 0.17 ± 0.12 to 1.18 ± 0.50 cm/s [Fig. 7(J&K)]. In comparison, SF6PP26 exhibited shorter mean time delays of 173 ± 189 ms without WES and 73 ± 53 ms under WES, with corresponding propagation velocities of 0.91 ± 0.50 and 1.77 ± 0.59 cm/s, respectively. Two-way ANOVA revealed a significant overall effect of hydrogel formulation on Ca^2+^ time delay (p = 0.0177) and propagation velocity (p = 0.0219). In contrast, the effects of WES were not statistically significant, although WES was associated with shorter mean time delays and higher apparent propagation velocities. These findings primarily support a formulation-dependent difference in Ca^2+^ propagation characteristics, with SF6PP26 exhibiting higher Ca^2+^ propagation velocity. In the present SF/PP system, the shorter Ca^2+^ delay and higher propagation velocity observed in SF6PP26 are consistent with a more electrically permissive PEDOT-containing hydrogel interface. When integrated with the contractility data, the Ca^2+^ imaging results suggest an association between formulation-dependent Ca^2+^ signalling characteristics and contraction-associated behaviour, rather than differences in beat frequency alone.

To determine whether the improved contractile behaviour and intracellular Ca^2+^ signalling observed on SF/PP hydrogels were accompanied by longer-term structural maturation, hiPSC-derived cardiomyocytes cultured on SF6PP12 and SF6PP26 were immunostained after 28 days of culture for sarcomeric α-actinin, cardiac troponin T (TnT), and Cx43. The presence of α-actinin-positive filamentous structures and TnT-positive contractile regions confirmed retention of cardiomyocyte identity under prolonged culture, while the appearance of striated α-actinin patterns in several regions indicated the formation of organised sarcomeric structures under both WES and no WES conditions [Fig. 8(A)]. Quantitative analysis of sarcomeric organisation showed that, after long-term culture, hiCMs on SF6PP12 and SF6PP26 hydrogels exhibited comparable α-actinin-positive Z-band spacing, with sarcomere lengths of approximately 1.70-1.75 μm under non-stimulated conditions [Fig. 8(C)]. Notably, WES significantly increased the sarcomeric length of hiCMs on both these hydrogel types to ∼2.1 μm (p < 0.01), approaching the sarcomere length typically reported for mature adult human cardiomyocytes, around 2.2 μm [51]. This comparable sarcomeric organisation was consistent with maintained spontaneous beating at day 28, with beat rates of approximately 21-28 beats/minute, and was further supported by longitudinal MUSCLEMOTION analysis comparing the day 8 contractile data shown in Fig. 7(E & G-I) with day 28 beating behaviour [Fig. S15, Supporting Information].

**Fig. 8:**
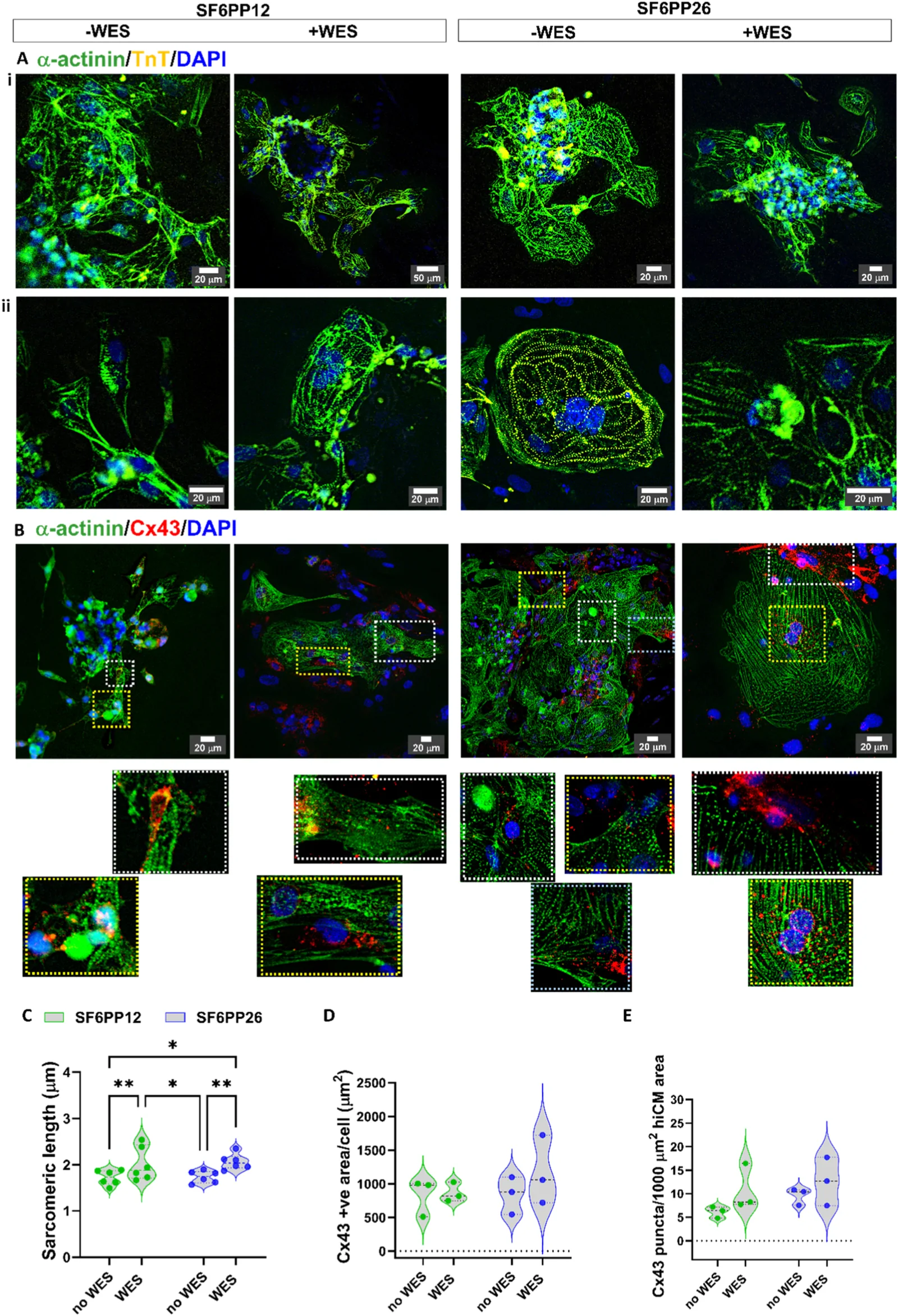
**Soft conductive SF/PP hydrogel formulations combined with wireless electrical stimulation (WES) promote sarcomeric organisation and Cx43-positive junctional maturation.** (A) Representative immunofluorescence images of hiPSC-derived cardiomyocytes cultured for 28 days on SF6PP12 and SF6PP26 hydrogels with or without WES, stained for α-actinin (green), cardiac troponin T (TnT; yellow), and DAPI (blue); A(i) shows low-magnification overview images of cardiomyocyte organisation across the hydrogel conditions, while A(ii) shows independent representative high-magnification images from the same experimental groups to visualise sarcomeric α-actinin/TnT organisation in greater detail. (B) Representative immunofluorescence images showing cardiomyocytes stained for α-actinin (green), connexin-43 (Cx43; red), and DAPI (blue). Dashed boxed regions are enlarged below to highlight Cx43-positive punctate structures within α-actinin-positive cardiomyocyte regions and near apparent cell-cell/peripheral contact zones. Quantification of (C) sarcomere length from α-actinin-positive Z-band spacing; (D) Cx43-positive area per cardiomyocyte within α-actinin-positive regions, and (E) Cx43 puncta density normalised to cardiomyocyte area. Data are presented as mean ± SD, n = 3 or more. *p < 0.05 and **p < 0.01.

Such contractile behaviour, Ca^2+^ signalling and structural measurements to assess cardiomyocyte functional maturation is consistent with previous hiCM maturation studies performed in conductive hydrogel and electrically conditioned engineered-myocardium systems. For example, Roshanbinfar et al. assessed enhanced hiPSC-CM maturation in PEDOT:PSS based conductive hydrogels using MUSCLEMOTION-derived contractility together with sarcomeric organisation and length [52]. Similarly, Ruan et al. evaluated functional maturation of electrically conditioned hiPSC-derived cardiac tissues using contractile function, Ca^2+^ signalling and Ca^2+^-handling protein expression [49]. In our work, functional electrical communication between the cardiomyocytes, as from day 8 intracellular Ca^2+^ signalling is directly associated with gap-junction-associated organisation; therefore, cells were also immunostained with Cx43 alongside sarcomeric α-actinin [Fig. 8(B)]. Cx43 appeared as punctate signal within α-actinin-positive cardiomyocyte regions, with several puncta located near the cell periphery and apparent cell-cell contact regions, as highlighted in the enlarged images [lower panel in Fig. 8(B)]. Quantification was restricted to α-actinin-positive cardiomyocyte regions in which sarcomeric organisation and DAPI-labelled nuclei could be clearly identified. This strategy minimised contribution from non-sarcomeric regions and allowed Cx43 signal to be assessed specifically within cardiomyocyte-associated areas.

Cx43 +ve area per cardiomyocyte remained comparable between non-stimulated and WES-treated SF6PP12 groups [Fig. 8(D)]. In SF6PP26, WES produced an upward shift in Cx43-positive area per cell, from 839 ± 279 μ m^2^ to 1167 ± 512 μ m^2^, although the difference did not reach statistical significance. Cx43 puncta density normalised to cardiomyocyte area increased under WES in both hydrogel formulations, rising approximately from 6 to 10 puncta/1000 μm^2^ to 12-14 puncta/1000 μm^2^ [Fig. 8(E)]. Two-way ANOVA revealed a significant overall effect of WES on Cx43 puncta density (p = 0.0305), although individual within-formulation comparisons were not statistically significant. Thus, while changes in Cx43-positive area were not statistically significant, puncta density exhibited a significant overall WES-associated increase across the two formulations.

Cx43 is the major ventricular gap-junction protein and is central to intercellular electrical coupling and impulse propagation between cardiomyocytes. Enhanced sarcolemmal Cx43 expression in iPSC-cardiomyocytes has been shown to improve intercellular coupling, increase sodium current, and enhance action-potential upstroke velocity [53]. Similarly, Li et al. reported that increased membrane localisation of Cx43 during iPSC-CM maturation was associated with increased conduction velocity [54]. In the present study, the observed Cx43 puncta organisation can be considered alongside the formulation-dependent differences in Ca^2+^ propagation [Fig. 7(J&K)], although a direct relationship was not established. Taken together, Fig. 7, Fig. 8 show that SF/PP hydrogels provide a mechanically robust bioelectronic interface for hiCM functional and structural organisation. SF6PP26, which combines higher PEDOT content, lower impedance, higher conductivity, and softer post-conditioning mechanics, supported faster Ca^2+^ propagation and stronger contraction-associated motion. Hydrogel electroactivity and capacitive WES provide complementary cues that support excitation-contraction-associated function, Ca^2+^ signal transmission, and structural maturation on SF/PP hydrogels that remain mechanically relevant to a remodelled cardiac microenvironment.

## 4 Conclusions

We demonstrate that pH-regulated SF/PP bioinks provide a printable electroconductive hydrogel bioink formulation for cardiac bioelectronic interfacing enabling WES to modulate cardiomyocyte function. pH neutralisation during SF/PP blending moderated rapid interactions between SF and the acidic PEDOT:PSS phase, thereby preventing premature heterogeneous gelation and extending the formulation into a reproducible extrusion window. Under these conditions, SF6PP12 and SF6PP26 exhibited shear-thinning behaviour, continuous filament extrusion, grid-level shape retention and post-deposition gel maturation through progressive β-sheet formation. We have also identified that PEDOT*GoP* incorporation altered the SF hydrogel network microarchitecture by increasing swelling capacity and pore area while reducing the compressive and storage moduli after aqueous conditioning. This resulted in bioelectronic interfaces with mechanical properties within the lower range of remodelled/fibrotic cardiac tissue, while maintaining high electrical conductivity and lower impedance at higher PEDOT*GoP* loading. The optimised formulations supported hiCM viability at the conductive hydrogel interface and, separately, enabled proof-of-concept extrusion of hiCF-laden tubular and grid constructs, demonstrating complementary cardiac biointerface and cell-laden processing capabilities.

These SF/PP hydrogels further enabled capacitive coupling based WES of hiCMs. A customised burst-modulated biphasic waveform was applied, consisting of a 10 kHz carrier delivered in 5 ms bursts at 2 Hz. This waveform was designed to enable capacitive signal transfer through the dielectric barrier while exposing the cells to an intermittent cardiac-relevant stimulation envelope. Importantly, the observed beat rates remained below the 2 Hz burst-envelope frequency, indicating that WES acted as a bioelectronic conditioning cue rather than frequency-locked pacing. SF6PP26, which combined higher PEDOT*GoP* loading, lower impedance and softer post-conditioning mechanics, supported stronger contractile behaviour, enhanced Ca^2+^ signalling and propagation velocity than SF6PP12. WES further increased the sarcomere length from approximately 1.70-1.75 μm under non-stimulated conditions to approximately 2.1 μm and was associated with higher overall Cx43 puncta density.

These findings establish pH-regulated SF/PP bioinks as crosslinker-free printable bioelectronic interfaces that integrate extrusion fidelity, cytocompatibility, electroactivity and capacitive coupling based WES. However, the current 10-15 min printing window remains a processing limitation, particularly for complex and multicellular cardiac biofabrication. Future work should therefore focus on extending the working time and further tuning SF network density, matrix compliance, cellular permissiveness and remodelling while preserving conductivity, filament stability, print fidelity and post-printing gel maturation. Such optimisation will be important for extending the platform to 3D hiCM-laden and multicellular cardiac bioprinting, together with direct electrophysiological measurements and disease-relevant injury models.

## CRediT authorship contribution statement

**Rajiv Borah:** Writing – review & editing, Writing – original draft, Visualization, Validation, Methodology, Investigation, Funding acquisition, Formal analysis, Data curation, Conceptualization. **Meenakshi Suku:** Writing – review & editing, Methodology, Investigation. **Dahnan Spurling:** Writing – review & editing, Methodology, Investigation. **Sara Corbezzolo:** Methodology, Investigation. **Jamie Brady:** Writing – review & editing, Methodology, Investigation, Formal analysis. **Tugdual Haffner:** Writing – review & editing, Methodology, Investigation. **Conor T. Buckley:** Writing – review & editing, Supervision. **Valeria Nicolosi:** Writing – review & editing, Supervision. **Michael G. Monaghan:** Writing – review & editing, Writing – original draft, Visualization, Validation, Supervision, Resources, Project administration, Methodology, Funding acquisition, Formal analysis, Conceptualization.

## Declaration of competing interest

The authors declare that they have no known competing financial interests or personal relationships that could have appeared to influence the work reported in this paper.

## Data Availability

Data will be made available on request.
