# Supplementary material for: Wireless bioelectronic stimulation enhanced by electroconductive silk bioink modulates human iPSC-cardiomyocyte contractility and structural organisation
Source: Mater Today Bio. 2026 Oct 24;41:103781. doi: 10.1016/j.mtbio.2026.103781 (PMC13770639; doi:10.1016/j.mtbio.2026.103781)
Supplement: Supplementary file 1 [file mmc17.docx]

**Supporting video file descriptions**

**MovieS 1:** Extrusion printing of SF6PP12 into a grid-like construct. Representative video showing pneumatic extrusion printing of the pH-neutralised SF6PP12 bioink at approximately 67 kPa. The formulation extruded continuously through the tapered nozzle (22G) and formed a grid-like construct with maintained filament continuity and visible pore definition after deposition.

**MovieS 2:** Representative video showing pneumatic extrusion printing of the pH-neutralised SF6PP26 bioink at approximately 45 kPa. The formulation was extruded through the tapered nozzle (22G) and deposited into a grid-like pattern; however, the printed strands showed visible spreading and broader filament deposition compared with sharper filament formation, consistent with the greater spreading tendency of SF6PP26 observed during printability assessment.

**MovieS 3:** Representative video showing pneumatic extrusion printing of the pH-neutralised SF6PP26 bioink at approximately 62 kPa. At this higher extrusion pressure, the deposited material showed pronounced strand broadening and spreading/over-deposition after nozzle contact, indicating reduced shape definition compared with the lower-pressure printing condition. This supports the identification of a lower pressure window as more suitable for SF6PP26 printing.

**MovieS 4:** Representative video showing pneumatic extrusion printing of the pH-neutralised SF6PP12 bioink into a freeform architecture at approximately 67 kPa. The formulation extruded continuously through the tapered nozzle (22G) and followed the intended freeform printing path, while retaining the overall deposited geometry after printing.

**MovieS 5:** Benchtop LED demonstration of burst-modulated capacitive signal transfer. Representative video showing transmission of a burst-modulated electrical signal through the capacitive-coupling setup used for wireless electrical stimulation (WES). A 10 kHz biphasic square-wave carrier (Vpp = 18 V) was delivered as 5 ms bursts at 2 Hz to the copper sheet beneath the petri dish, while the output response was monitored using an LED and oscilloscope connected to the conductive receiver interface. The LED illumination during each burst, together with the corresponding oscilloscope signal, provides visual confirmation that discrete burst packets can be transmitted through the capacitive setup.

**MovieS 6-9:** Representative bright-field videos (4X) showing beating hiCMs cultured on SF6PP12 and SF6PP26 hydrogels with or without WES on day 8. Movies S6 and S8 show SF6PP12 and SF6PP26, with no electrical stimulation, respectively; while Movies S7 and S9 show SF6PP12 and SF6PP26 with electrical stimulation, respectively. −WES and +WES conditions, respectively. These videos correspond to the fields used for MUSCLEMOTION-based analysis of beat rate, relative contraction amplitude and mean peak contraction speed in **Figure 7[E & (G-I)]**.

**MovieS 10-13:** Representative live Cal-520 imaging videos of intracellular Ca^2+^ transients in hiCMs cultured on SF/PP hydrogels on day 8. Movie S10 and Movie S12 show SF6PP12 and SF6PP26 without electrical stimulation, respectively; while Movie S11 and Movie 13 show SF6PP12 and SF6PP26 with electrical stimulation, respectively. The videos show repeated Ca^2+^-associated fluorescence transients across the imaged cardiomyocyte regions. Compared with the non-stimulated groups, WES-treated groups show more coordinated fluorescence transients, with SF6PP26 +WES displaying the most pronounced and spatially organised Ca^2+^ flow. These movies correspond to the representative fields and ROI-based Ca^2+^ transient analyses shown in **Figure 7(F,J,K)**.

**Movie S14:** Representative video showing a printed SF6PP26 hydrogel construct subjected to unconfined dynamic cyclic compression between parallel platens at 25% compressive strain and a frequency of 1 Hz. The construct underwent repeated deformation and recovery while maintaining its overall macroscopic integrity, without visible fracture, fragmentation, or layer separation during the recorded cycles.

**Movie S15:** Representative video showing freshly deposited SF6PP26 in the pre-gelation stage on chicken breast tissue mounted in a custom-built, Arduino-controlled electromechanical tensile deformation system. The tissue was subjected to cyclic tensile deformation at 25% strain and 1 Hz. The deposited SF6PP26 remained attached to the tissue surface and deformed with the underlying tissue without visible detachment or delamination during the recorded cycles.

**Movie S16:** Representative video showing the same SF6PP26 deposited chicken breast tissue presented in Movie S15 after gelation by incubation at 37°C for 30 minutes under repeated cyclic tensile deformation at 25% strain and 1 Hz (Arduino-controlled electromechanical tensile deformation system). The gelled SF6PP26 remained attached to the tissue surface and deformed with the underlying tissue while retaining its overall deposited structure, without visible macroscopic detachment or displacement during the recorded cycles.
