# Supplementary material for: Wireless bioelectronic stimulation enhanced by electroconductive silk bioink modulates human iPSC-cardiomyocyte contractility and structural organisation
Source: Mater Today Bio. 2026 Oct 24;41:103781. doi: 10.1016/j.mtbio.2026.103781 (PMC13770639; doi:10.1016/j.mtbio.2026.103781)
Supplement: Supplementary file 2 [file mmc18.docx]

**Supporting Information**

**Wireless bioelectronic stimulation enhanced by electroconductive silk bioink modulates human iPSC-cardiomyocyte contractility and structural organisation**

Rajiv Borah^1,2^ , Meenakshi Suku^1,2,3^, Dahnan Spurling^4,5^, Sara Corbezzolo^1,2^**,** Jamie Brady^1,2^**,** Tugdual Haffner^1,2^ , Conor T. Buckley^1,2^ , Valeria Nicolosi^4,5^, Michael G. Monaghan^1,2,3, *^

^1^Discipline of Mechanical, Manufacturing and Biomedical Engineering, School of Engineering, Trinity College Dublin, Dublin 2, Ireland

^2^Trinity Centre for Biomedical Engineering, School of Engineering, Trinity College Dublin, Dublin 2

^3^CÚRAM, Centre for Research in Medical Devices, National University of Ireland, H91 W2TY Galway, Ireland

^4^Centre for Research on Adaptive Nanostructures and Nanodevices (CRANN), Trinity College Dublin, Dublin, Dublin 2, Ireland

^5^School of Chemistry, Trinity College Dublin, Dublin, Dublin 2, Ireland

*E-mail: [monaghmi@tcd.ie](mailto:monaghmi@tcd.ie)

**Methods**

**S1. UV-Vis based spectroscopy for gelation study of SF/PP formulations**

Early SF/PP gelation kinetics as a function of pH adjustment were assessed by monitoring turbidity using a UV-Visible absorbance-based method adapted from our previously reported protocol.^1^ Briefly, freshly prepared SF6PP12 and SF6PP26 formulations were adjusted using either 1 or 2 µL of 2 M NaOH per mL of formulation. Immediately after mixing, 200 µL of each formulation was transferred into a covered 96-well plate and incubated at 37°C in a temperature-controlled microplate reader (Synergy HT Multi-Mode Microplate Reader, BioTek Instruments, USA). Absorbance at 550 nm (A550) was recorded at 5 minutes intervals for 30 minutes to monitor the increase in light scattering associated with SF/PP aggregation and early network formation. The measurement period was limited to 30 minutes because several conductive formulations subsequently exceeded the reliable absorbance range. To account for the intrinsic optical absorbance of PEDOT:PSS, matched PEDOT*GoP* controls containing the corresponding PEDOTGoP concentration (0.12 and 0.26 wt%) and NaOH dose but without SF were measured in parallel, and their A550 values were subtracted from those of the corresponding SF/PP formulations. Pure 6 wt% SF (SF6) was included as a non-conductive control. Non-neutralised SF6PP12 and SF6PP26 formulations were not included in the quantitative turbidity analysis because rapid heterogeneous gelation immediately after mixing prevented reproducible transfer into the microplate. All measurements were performed in triplicate (n = 3), and data are presented as mean ± SD.

**S2. PEDOT:PSS leaching assessment by UV-Vis spectroscopy**

PEDOT:PSS leaching from SF/PP hydrogels was quantified using a UV-Vis absorbance-based method adapted from previous studies evaluating PEDOT:PSS release and aqueous stability.^2, 3^ SF6PP12 and SF6PP26 hydrogels (200 µL; n = 5) were prepared in a 48 well plate and incubated at 37°C with 500 µL of either 1× PBS (pH 7.4) or PBS containing protease XIV (2 U/mL). PEDOT:PSS-free SF6 hydrogels were incubated under the corresponding conditions and used as background controls. At defined time points (days 1, 5, 10, 15 and 20), the incubation medium was completely collected and replaced with 500 µL of fresh PBS or protease-containing solution, as appropriate. A 200 µL aliquot of each collected supernatant was transferred to a 96 well plate for absorbance measurement.

PEDOT:PSS-equivalent concentration was determined from absorbance at 850 nm using a calibration curve prepared from serial dilutions of PEDOT*GoP*. Absorbance at 850 nm has been attributed predominantly to localized polaron states of oxidised PEDOT and has been used to monitor the aqueous stability of PEDOT:PSS and GOPS-modified PEDOT:PSS previously.^2^ Use of the near-infrared region also minimises interference from SF and protein/peptide-derived degradation products that exhibit stronger absorption in the UV region.

**S3. Microenvironmental temperature monitoring during WES**

Microenvironmental temperature was monitored to assess potential heating during capacitive-coupling wireless electrical stimulation (WES). The measurements reproduced the physical configuration used for WES of hiPSC-derived cardiomyocytes, with SF/PP hydrogels deposited on glass coverslips and immersed in RPMI-based culture medium, but without cells. Four thermocouple probes connected to a multi-channel thermocouple thermometer (HT-9815, HTI Instrument, Dongguan, China) were positioned at predefined locations within the culture setup. SF6PP12 and SF6PP26 hydrogels were evaluated under WES and no-WES conditions in the same incubator environment. WES was applied using the stimulation waveform described in **Section 2.16** of the main manuscript: an 18 Vpp (±9 V), 10 kHz biphasic carrier delivered in 5 ms bursts at 2 Hz, corresponding to a 1% duty cycle. Temperature was recorded at defined intervals for up to 48 hours. The experimental configuration and temperature profiles are presented in **Figure S14(A,B)**.

**S4. Electrothermal finite-element modelling**

Finite-element modelling was performed using COMSOL Multiphysics 6.4 (COMSOL AB, Stockholm, Sweden) to evaluate the electrical-field distribution and WES-associated electrothermal response of the experimental culture configuration. A three-dimensional model was constructed comprising a cylindrical copper transmitter electrode (radius: 15 mm), a 1 mm polystyrene dish layer, a 0.13 mm glass coverslip, a cylindrical RPMI-based culture-medium domain (radius: 15 mm; height: 8 mm), and a centrally positioned SF/PP hydrogel receiver (radius: 5 mm; height: 2.55 mm; volume: approximately 200 µL). Adjacent domains shared conformal interfaces. A representative electrical conductivity of 3.5 S m⁻¹ was assigned to the SF/PP receiver for the electrothermal model. The model geometry is shown in **Figure S14(C)**, and the electrical and thermal material properties used in the simulations are summarised in **Table S1**.

**S4.1 Electrical formulation**

Electrical behaviour was solved using the Electric Currents interface under the quasi-electrostatic approximation. The electric field E, electric displacement D and total current density J were related to the scalar electric potential V according to

$$E = -\nabla V, D = \varepsilon_{r}\varepsilon_{0}E, J = \sigma E + \frac{\partial D}{\partial t}$$

$$\nabla\cdot J = 0$$

A voltage-terminal condition, V = V_in(t), was imposed on the underside of the copper transmitter electrode (term1), while the top surface of the culture-medium domain was grounded (gnd1; V = 0). All remaining exterior electrical boundaries were insulating, n · J = 0. Normal current was conserved across each internal interface.

The experimentally applied waveform was represented as an 18 V peak-to-peak (±9 V) biphasic square-wave carrier at 10 kHz, delivered in 5 ms bursts at a repetition frequency of 2 Hz. Each 500 ms envelope period therefore contained a 5 ms active interval and a 495 ms inactive interval, corresponding to a duty fraction D = 0.01. A 1 µs 10-90% rise/fall time was applied to regularise the voltage transitions. A time-dependent electrical solution was computed from 0 to 7 ms, comprising a 1 ms pre-stimulation baseline, one complete 5 ms burst and a 1 ms post-burst interval

**S4.2 Harmonic losses**

To resolve carrier-associated losses without explicitly time-stepping every 10 kHz cycle during the long thermal simulation, the ideal biphasic carrier was represented by the first 20 odd Fourier harmonics:

$$V\left( t \right) = \sum_{m = 0}^{19} \left[ \frac{4V_{pk}}{\left( 2m+1 \right)\pi} \right]\sin\left[ \left( 2m+1 \right)\omega_{0}t \right]$$

where Vₚₖ = 9 V and ω₀ = 2π(10 kHz). The retained harmonic orders were n = 1, 3, ..., 39, giving frequencies from 10 to 390 kHz and peak harmonic amplitudes Vₙ = 4Vₚₖ/(πn). For each harmonic, the cycle-averaged volumetric electrical-loss density was evaluated as

$$q_{sum} = \sum_{n}^{\in} \left( 1,3,5,\ldots,39 \right)q_{n}, q_{n} = \frac{1}{2}Re\left\{ J_{n}\cdot E_{n}^{*} \right\}$$

The harmonic loss fields were summed spatially and passed to the Heat Transfer interface as the WES heat-source field.

**S4.3 Electrothermal effect-** **Transient heat-transfer model**

Temperature evolution was governed by the transient heat equation

$$\rho C_{p}\frac{\partial T}{\partial t} = \nabla\left( k\nabla T \right) + Q_{WES} \left( 5 \right)$$

For the duty-averaged long-duration study, the volumetric source was defined as

$Q_{WES} = Dq_{sum} \left( 6 \right)$

with D = 0.01.

All domains were initialised at 310.15 K. A convective heat-flux condition was applied to the external surfaces,

$$-k\nabla T \cdot n = h\left( T-T_{\infty} \right) \left( 7 \right)$$

with h = 5 W m⁻² K⁻¹ and T∞ = 310.15 K, representing the incubator environment. Temperature and normal heat flux were continuous across all internal interfaces. Transient heat transfer was simulated for 48 h, representing one interval between medium changes during the seven-day WES experiment. Medium replacement, which occurred experimentally every two days, was not represented as a discrete event. A stationary thermal solution was also calculated to estimate the limiting temperature plateau.

**S4.4 Mesh and numerical solution**

The geometry was discretised using a physics-controlled fine tetrahedral mesh. Time-dependent solutions were computed using a relative tolerance of 10^-3^, a maximum time-stepping order of 2 and, for the 48 hours thermal study, a maximum internal time step of 30 minutes.

**Table S1.** Electrical and thermal properties used in the FEM model

| Domain | σ (S m⁻¹) | εᵣ | ρ (kg m⁻³) | Cₚ (J kg⁻¹ K⁻¹) | k (W m⁻¹ K⁻¹) |
| --- | --- | --- | --- | --- | --- |
| Copper | 5.98$\cdot{10}^{7}$ | 1 | 8960 | 385 | 400 |
| Polystyrene | ~0 | 2.6 | 1050 | 1200 | 0.12 |
| Glass | ~0 | 4.2 | 2210 | 730 | 1.4 |
| Culture medium | 1.5 | 60 | 1010 | 4150 | 0.62 |
| SF/PP hydrogel | 3.5 | 80 | 1010 | 4000 | 0.6 |

**Figure S1:** Visual assessment of pH-dependent SF/PP gelation behaviour. Representative inversion test images showing SF/PP formulation behaviour under (A) low-pH and (B) pH-neutralised conditions. Under low-pH conditions, SF/PP mixtures showed rapid, heterogeneous gelation/phase separation within 2 minutes (at room temperature). In contrast, pH neutralisation produced a more homogeneous SF/PP formulation with delayed gelation (referred as partial gelation stage throughout this work and used for extrusion printing), followed by mature gel formation after incubation at 37˚C for 25 minutes.

**Figure S2: NaOH-dependent modulation of early SF/PP gelation kinetics.** Data are presented as mean ± SD, n = 3.

**Figure S3:** Time-dependent pyrene micropolarity index (I_373_/I_383_) for pure water (dH₂O), PEDOT:PSS, PEDOTGoP and SF controls over 70 minutes [5 µM pyrene (Py) was used for each groups]. dH₂O, PEDOT:PSS and PEDOTGoP showed only minor changes in the micropolarity index, whereas SF showed an early transient increase followed by a decrease towards baseline. This indicates that the time-dependent pyrene response in SF-containing formulations is mainly associated with changes in the local SF microenvironment rather than pyrene signal drift or PEDOTGoP alone.

**Figure S4: FT-IR and EDX characterisation of GOPS-modified PEDOT:PSS and its incorporation into the SF/PP composite.** (A) Representative SEM image of freeze-dried unmodified PEDOT:PSS and the corresponding EDX spectrum acquired from the highlighted region, showing predominantly C, O and S, with no detectable Si signal. (B) Representative SEM image and corresponding EDX spectrum of GOPS-modified PEDOT:PSS (PEDOTGoP). (C) Comparative FT-IR spectra of PEDOT:PSS and PEDOTGoP. (D) EDX spectrum of the SF6PP12 composite, with the corresponding SEM image and analysed region shown in the inset, demonstrating detectable Si after incorporation of PEDOTGoP into the SF matrix. The boxed regions in the SEM images indicate the areas used for EDX acquisition. Au signals originate from the conductive coating used for SEM/EDX analysis.

**Figure S5:** Gaussian peak deconvolution of FT-IR amide I spectra during SF/PP gelation. Gaussian peak deconvolution of the amide I region of pH-neutralised SF6PP12 and SF6PP26 formulations at different gelation stages: (A & B) before gelation (BG); (C & D) partial gelation (PG) and (E & F) after gelation (AG), with the corresponding second-derivative spectra shown alongside each fit. Negative minima in the second-derivative spectra were used to guide candidate component peak positions for curve fitting. The black line represents the experimental absorbance spectrum, the yellow line the cumulative fitted spectrum, The coloured dotted curves represent fitted secondary-structure contributions assigned to β-sheets (B; 1695-1700 cm^-1^; 1610-1635 cm^-1^), turns (T; 1666-1685 cm^-1^), α-helices (A; 1650-1665 cm^-1^), random coils (R; 1635-1645 cm^-1^) and side chains (SC; 1600-1610 cm^-1^).

**Figure S6:** Preliminary printability assessment with low SF concentration. Representative images showing (A) extrusion of SF/PP formulation through a tapered nozzle (20G) with 3 wt% SF and 0.26 wt% of PEDOTGoP and (B) poor self-standing filament stability after deposition on a filament-collapse platform. Although the 3 wt% SF formulation could be extruded, the deposited material lacked sufficient structural integrity to maintain self-standing fidelity, supporting the selection of 6 wt% SF for subsequent SF/PP bioink development.

**Figure S7:** Remaining mass of SF6, SF6PP12 and SF6PP26 hydrogels during in vitro enzymatic degradation in PBS containing 2 U/mL protease XIV at 37˚C over 30 days. Hydrogel samples were incubated individually in protease-containing PBS, with the enzyme solution replaced every 5 days. Remaining mass was calculated relative to the initial hydrogel mass at each time point. All formulations showed gradual protease-mediated mass loss over time, confirming the enzymatic degradability of the silk-based hydrogel networks. Data are presented as mean ± SD.

**Figure S8: Cytoskeletal morphology of hiPSC-derived cardiac cell cultures on media-conditioned SF/PP hydrogel surfaces.** Representative fluorescence images of cells cultured for 7 days on media-preconditioned SF6PP12 and SF6PP26 hydrogels. F-actin was visualised using phalloidin staining (red), with nuclei counterstained using DAPI (blue). Low-magnification images (top; scale bar = 100 µm) show the overall distribution and attachment of cells on the hydrogel surfaces. The dashed boxes indicate representative regions shown at higher magnification below (scale bar = 20 µm), demonstrating adherent and spread cellular morphologies with prominent F-actin organisation and cytoskeletal bundles. Localised cell clustering was also observed, particularly on SF6PP26. As these hiPSC-derived cardiac cultures were not subjected to lactate-based metabolic purification and therefore contained cardiomyocytes together with non-cardiomyocyte populations, the images are presented as a qualitative assessment of overall cellular morphology and cytoskeletal organisation rather than cardiomyocyte-specific structural maturation.

**Figure S9:** Representative live/dead images of human iPSC-derived cardiac fibroblasts (hiCFs) embedded within SF6 hydrogel at 12 and 72 hours. Live cells are shown in green and dead cells in red. Qualitatively, the images indicate a time-dependent reduction in cell survival within the pure SF6 hydrogel, highlighting the influence of formulation-specific network structure on cell compatibility. White arrowheads indicate autofluorescence (red) from silk matriz Scale bars: 500 µm.

**Figure S10:** Cumulative PEDOT:PSS-equivalent release from SF/PP hydrogels during prolonged aqueous incubation and enzymatic degradation**.** Data are presented as mean ± SD (**n = 5**).

**Figure S11:** Electrochemical impedance behaviour and Nyquist fitting of SF/PP hydrogel bioinks. Bode plots showing frequency-dependent impedance magnitude and phase response of (A) SF6PP12 and (B) SF6PP26 hydrogels measured in PBS. Corresponding Nyquist plots with fitted curves for (C) SF6PP12 and (D) SF6PP26. The Bode spectra show the frequency-dependent electrochemical response of the conductive hydrogel interfaces, while the fitted Nyquist plots were used to assess interfacial charge-transfer behaviour.

**Figure S12:** Room temperature electrical conductivity of SF6PP12 and SF6PP26 lyophilised scaffolds measured using two probe method. Data are mean ± SD (n=4).

**Figure S13: Expanded validation of the WES waveform corresponding to Figure 7(D).** (A) Burst timing showing 5 ms ON and 495 ms OFF periods, corresponding to 2 Hz repetition and a 1% duty cycle. (B) Complete 5 ms stimulation burst at the function-generator output, comprising 50 cycles of an 18 Vpp (±9 V), 10 kHz biphasic carrier. (C) Expanded view of the 10 kHz carrier waveform and discrete sampling points.

**Figure S14:** **Experimental and computational assessment of thermal effects during capacitive-coupling wireless electrical stimulation (WES).** (A) Representative experimental setup for multi-point thermocouple monitoring of the culture microenvironment during WES. (B) Temperature profiles measured at four thermocouple locations for SF/PP formulations under WES and no-WES conditions over the stimulation period; data are presented as mean ± SD across the four monitored positions. Following initial thermal equilibration, temperatures remained relatively stable during prolonged WES. (C) Three-dimensional finite-element model reproducing the experimental WES configuration, comprising the copper transmitter electrode, dielectric layers, cell culture medium, and centrally positioned SF/PP hydrogel receiver. (D) FEM-predicted volume-average thermal response over 48 h, showing an increase from the initial **37˚C** to approximately **38.2˚C (ΔT ≈ 1.2 °C),** approaching a plateau within approximately **4 h**ours and remaining stable thereafter. (E) Spatial distributions of the predicted temperature change at 0, 1, 2, and 4 hours.

**Figure S15:** Longitudinal MUSCLEMOTION analysis of hiCM contractile behaviour. (A) Representative day 28 bright-field images and matched MUSCLEMOTION contraction traces from selected regions of interest in hiCM cultures on SF6PP12 and SF6PP26 hydrogels with and without WES (Scale bar= 50 μm). (B) Quantitative analysis compares day-8 MUSCLEMOTION data shown in Figure 7 of the main manuscript with day-28 spontaneous beating behaviour, including beat rate, contraction amplitude and peak contraction speed, confirming that contractile activity was maintained up to day 28 across SF/PP groups.

**References**

(1) Bhunia, B. K.; Mandal, B. B. Exploring gelation and physicochemical behavior of in situ bioresponsive silk hydrogels for disc degeneration therapy. *Acs Biomater Sci Eng* **2019**, *5* (2), 870–886.

(2) Håkansson, A.; Han, S.; Wang, S.; Lu, J.; Braun, S.; Fahlman, M.; Berggren, M.; Crispin, X.; Fabiano, S. Effect of (3‐glycidyloxypropyl) trimethoxysilane (GOPS) on the electrical properties of PEDOT: PSS films. *Journal of Polymer Science Part B: Polymer Physics* **2017**, *55* (10), 814–820.

(3) Roshanbinfar, K.; Schiffer, M.; Carls, E.; Angeloni, M.; Koleśnik‐Gray, M.; Schruefer, S.; Schubert, D. W.; Ferrazzi, F.; Krstić, V.; Fleischmann, B. K. Electrically conductive collagen‐PEDOT: PSS hydrogel prevents post‐infarct cardiac arrhythmia and supports hiPSC‐cardiomyocyte function. *Adv Mater* **2024**, *36* (28), 2403642.
